# Supplementary material for: Climate change favours large seasonal loss of Arctic ozone
Source: Nat Commun. 2021 Jun 23;12:3886. doi: 10.1038/s41467-021-24089-6 (PMC8222337; doi:10.1038/s41467-021-24089-6)
Supplement: Supplementary file 1 — Supplementary Info [file 41467_2021_24089_MOESM1_ESM.pdf]

Supplementary Information - The influence of climate change on chemical loss of ozone in the Arctic stratosphere by von der Gathen et al.

This supplement contains 20 additional figures and four additional tables that support material in the paper. Below, we accompany each figure and table with only a brief description since all elements of this SI are described in either the Main article or Methods section.

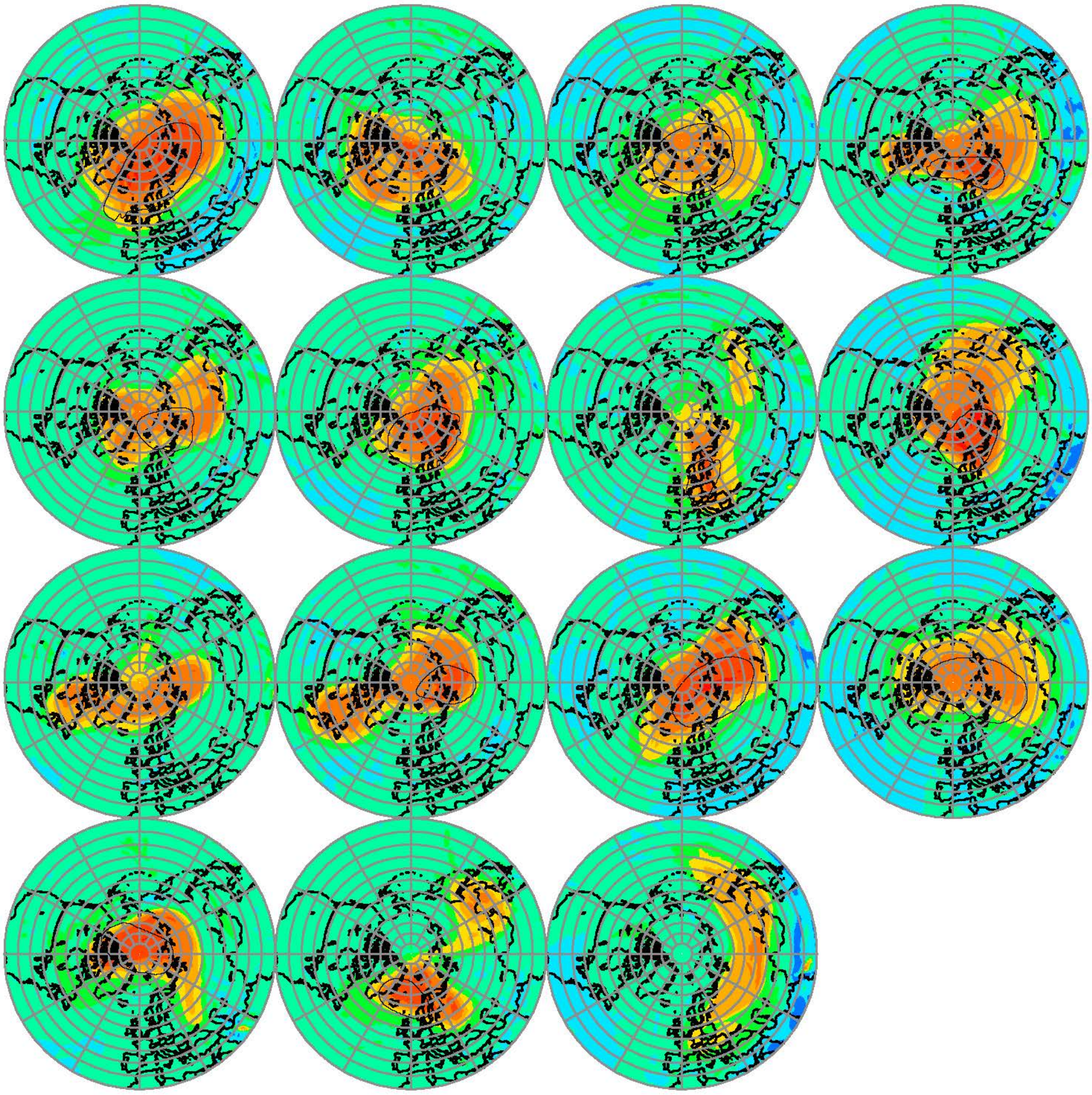

Supplementary Figure 1a. PV and Temperature, EC-Earth3. Maps of nPV on 1 February of 1960, followed by 1 February of every 10 years until 2100, at the 475 K potential temperature surface, from the EC-Earth3 GCM. The archived temperature field has been adjusted by a constant offset specific to each model, so that the overall magnitude of $\mathrm{PFP}^{\mathrm{LM}}$ in the GCM matches the observed magnitude of $\mathrm{PFP}^{\mathrm{LM}}$, over the modern satellite era. The area for which temperature is less than T_{NAT} is indicated by the thin, continuous black line. Here, T_{NAT} is based on the assumption of constant stratospheric H_2O of 4.6 ppmv.

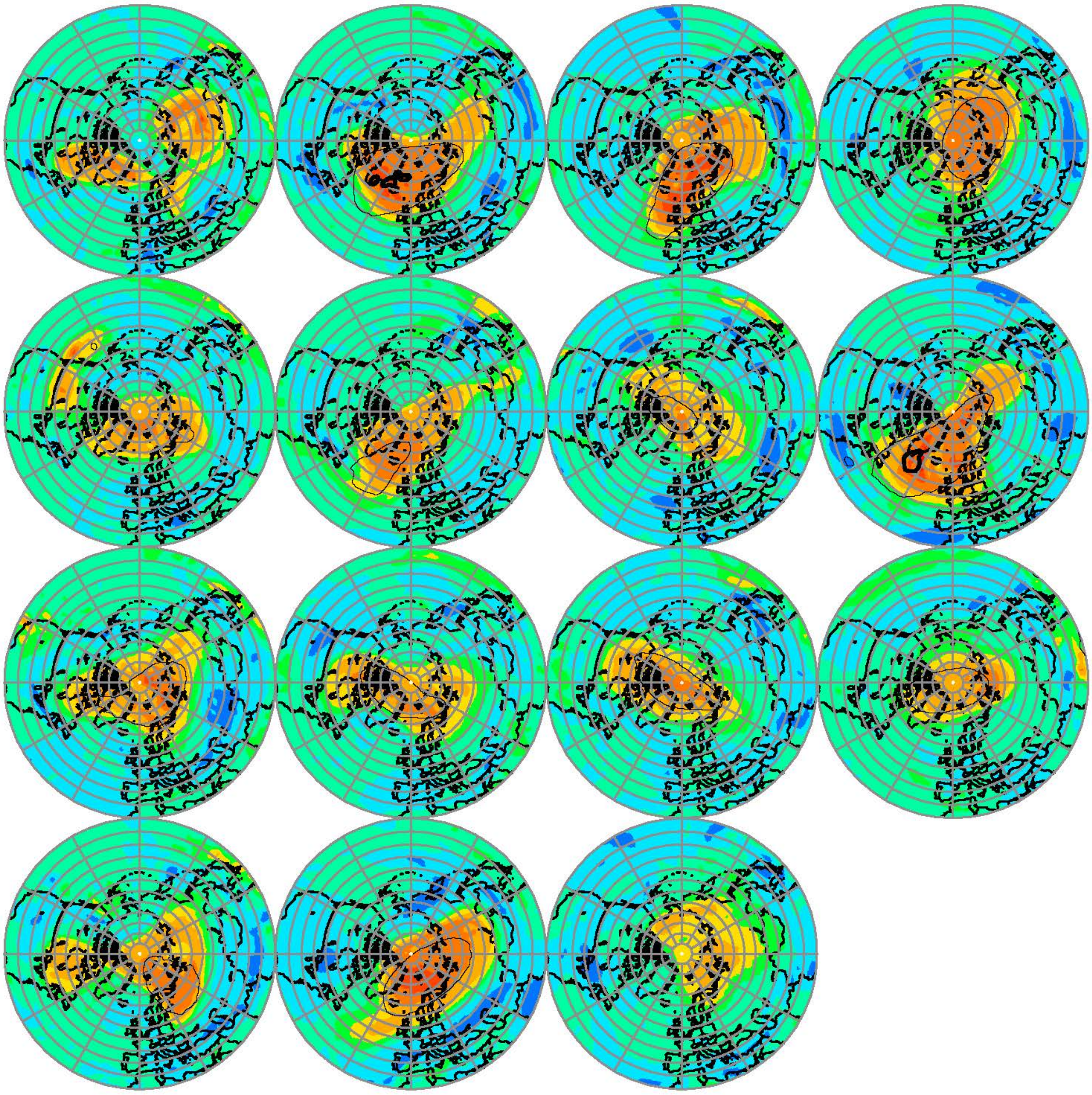

Supplementary Figure 1b. PV and Temperature, MIROC6. Same as Supplementary Fig. 1a, for output of the MIROC6 GCM, except for this model, Arctic temperature also drops below the frost point of water ice. Here, the thick black line denotes the area for which temperature is less than $T_{\rm ICE}$.

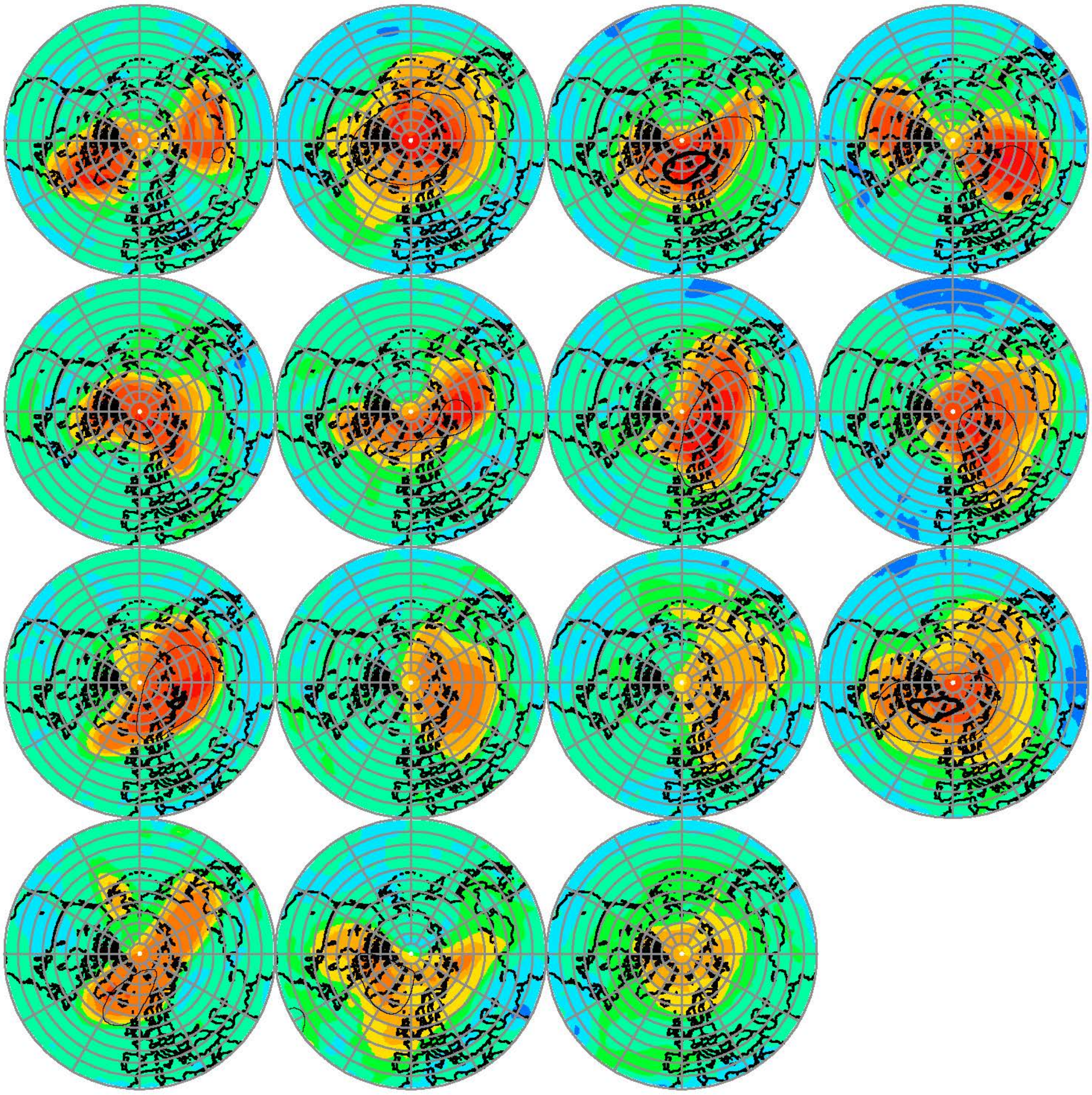

Supplementary Figure 1c. PV and Temperature, MPI-ESM1-2-LR. Same as Supplementary Fig. 1b, for output of the MPI-ESM1-2-LR GCM.

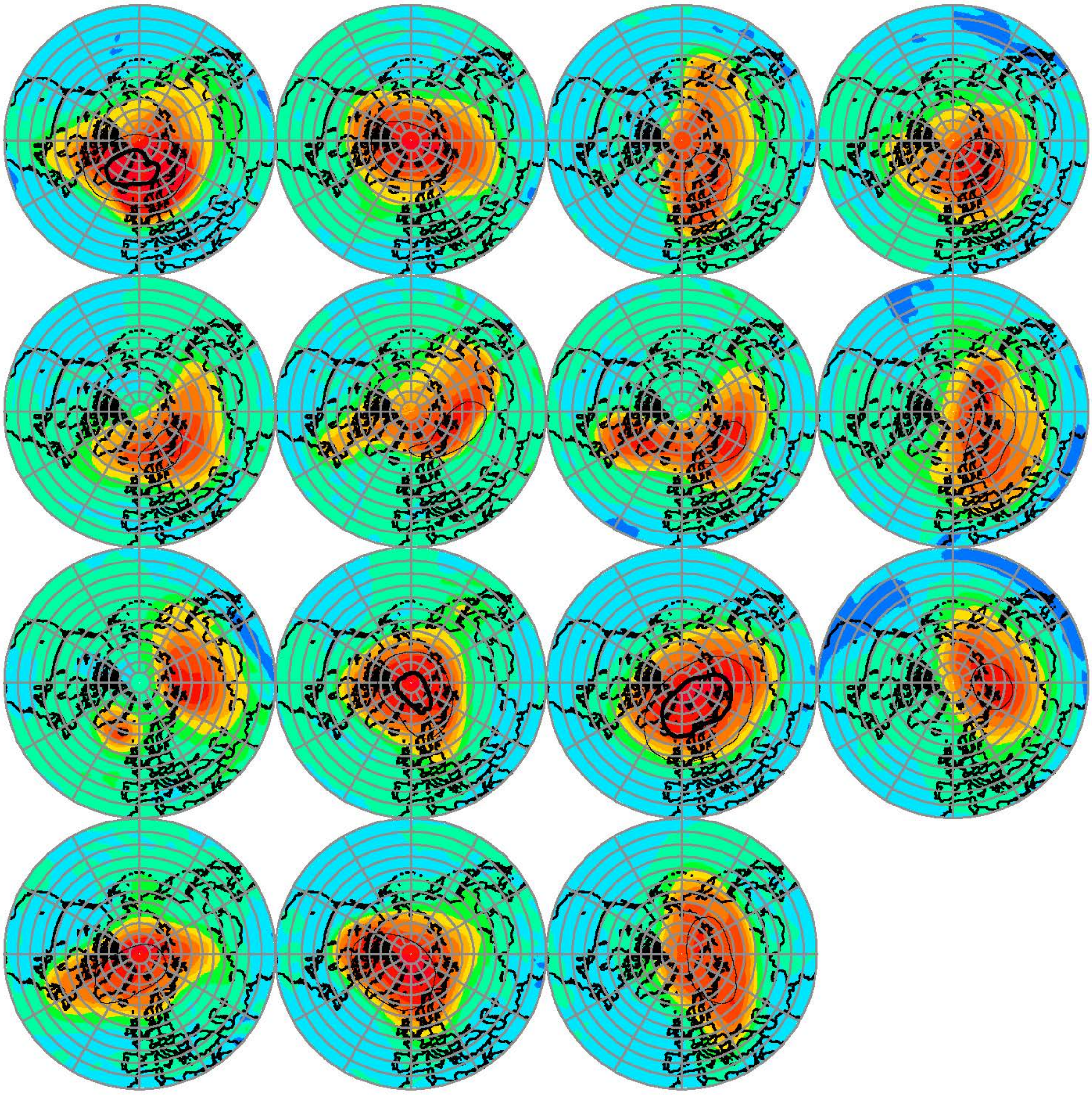

Supplementary Figure 1d. PV and Temperature, UKESM1-0-LL. Same as Supplementary Fig. 1b, for output of the UKESM1-0-LL GCM.

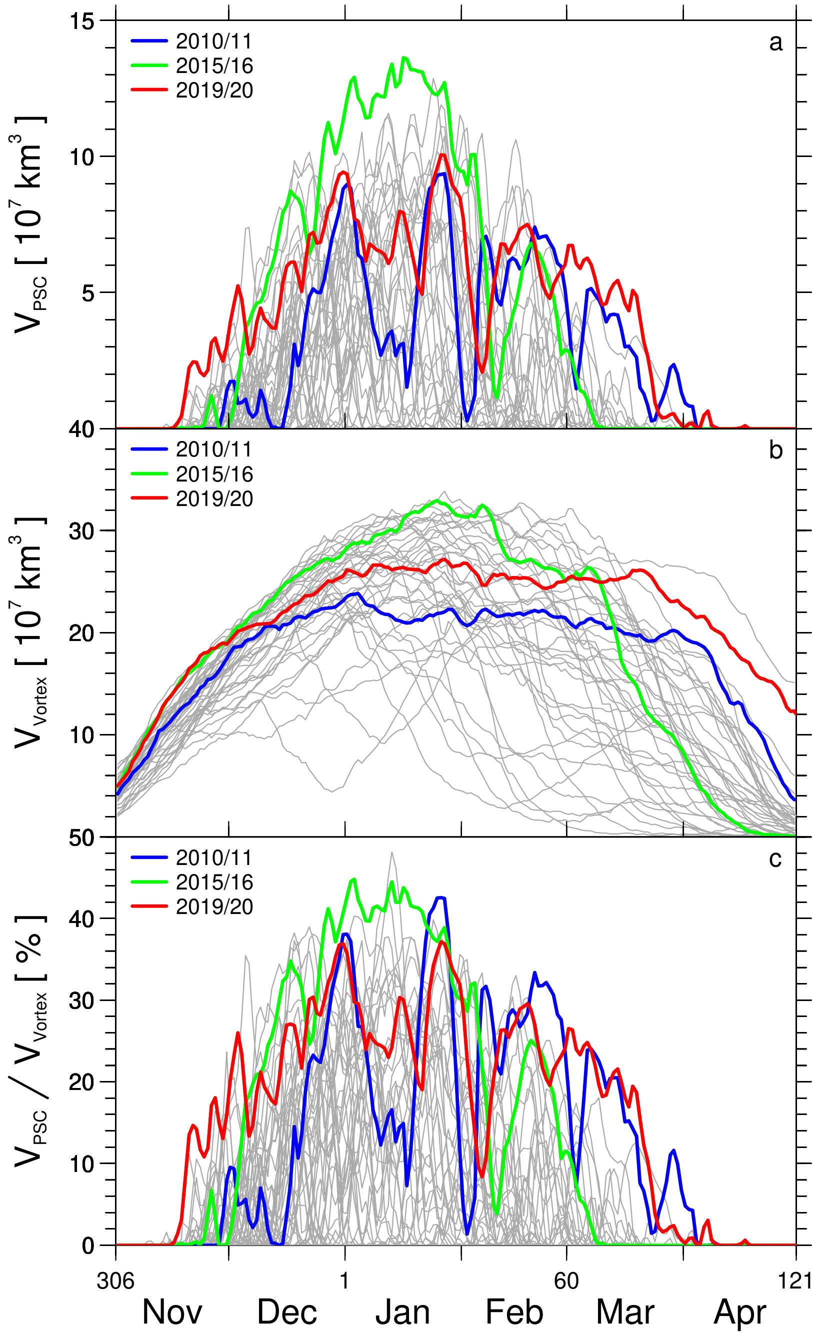

Supplementary Figure 2. V_{PSC} **and** V_{VORTEX}**. a-c,** Temporal evolution of the volume of air with temperature below the threshold for formation of PSCs inside Arctic stratospheric vortex (**a**), the volume of the Arctic stratospheric vortex (**b**), and the ratio of these two terms (**c**) based on analysis of data from ERA5/ERA5.1 (ref.[1]) over 1980-2020 for the individual years (grey). Values for particularly cold Arctic winters, including the record setting winter of 2019/2020 (red), are highlighted. The area under each curve shown in (**c**) defines the PSC formation potential (PFP) for a particular winter.

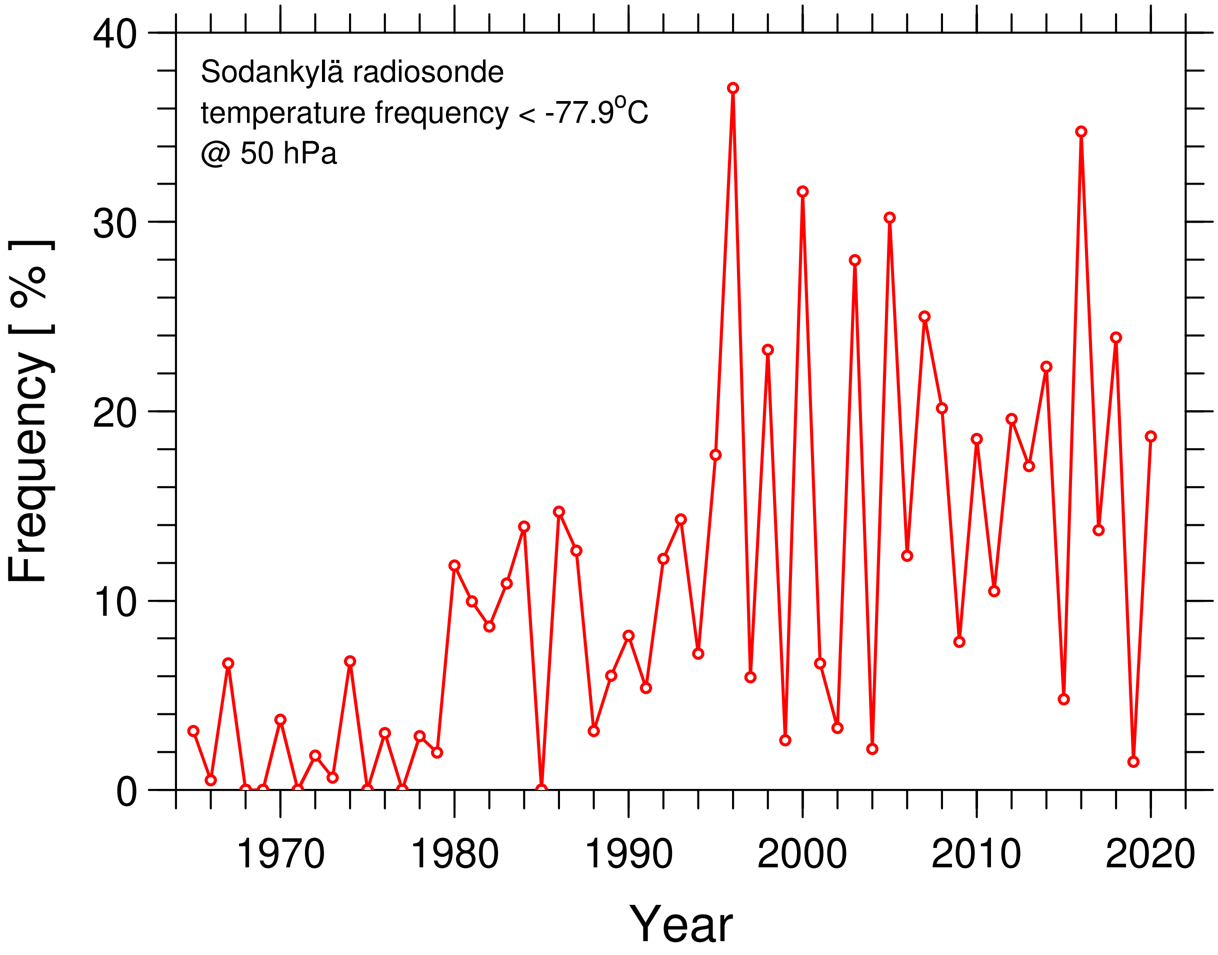

Supplementary Figure 3. PSC temperature record, Sodankylä. Percentage of observations of temperature below −77.9°C at 50 hPa over the months of December (prior year) and January, February, and March (indicated year) from regular radiosondes launched from Sodankylä, Finland (67° 22´ N, 26° 39´ E) (ref.[2]).

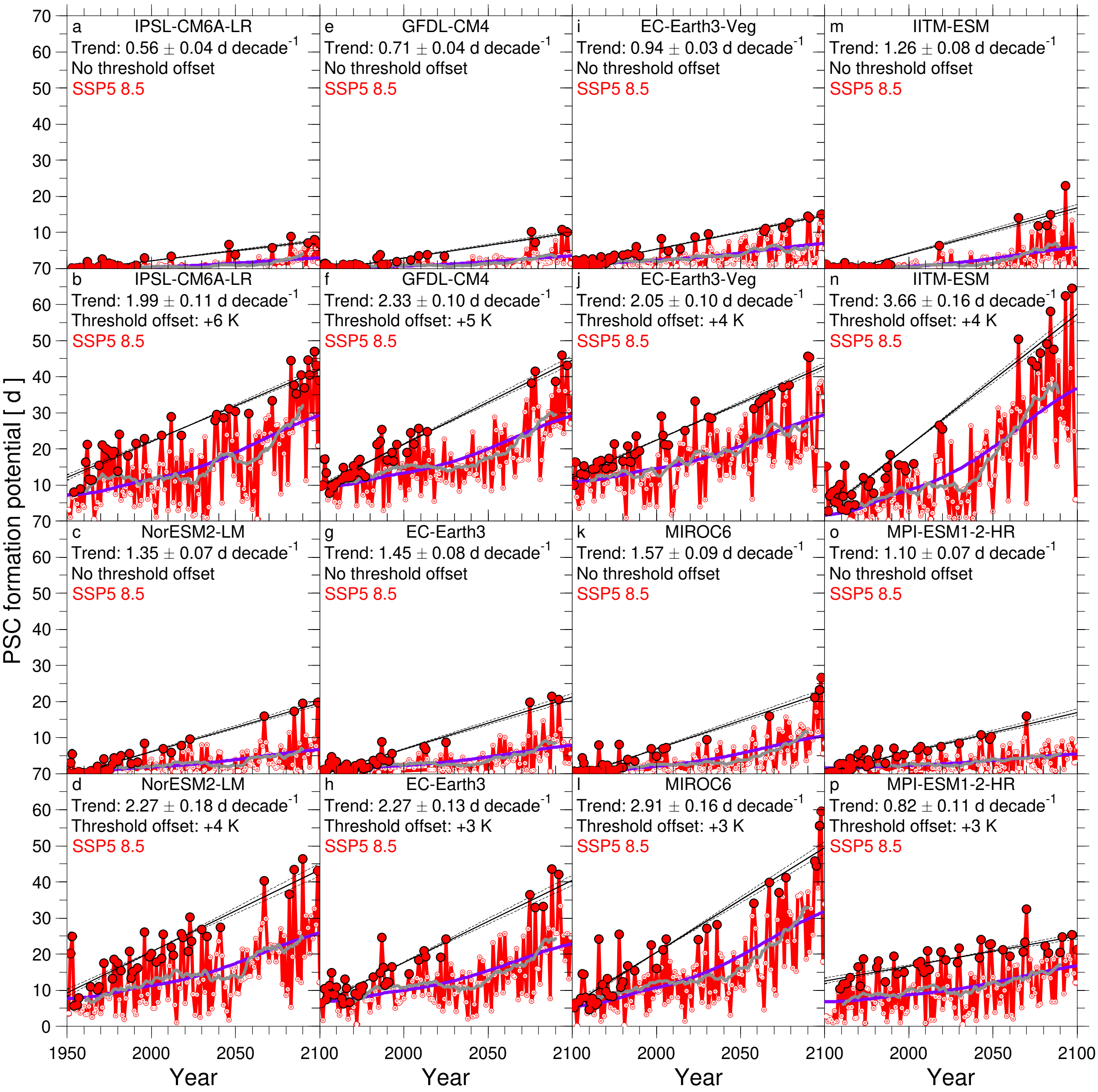

Supplementary Figure 4a. PSC Formation Potential (PFP) from the first eight CMIP6 GCMs for the SSP5-8.5 scenario, with and without adjustment of the temperature for formation of PSCs. a-p, Time series of PFP for eight CMIP6 GCM simulations, without (upper panel of each pair) and with (bottom panel) an adjustment of the formation temperature of PSCs. Each panel also indicates the coldest winters in the record (local maxima) relative to trend in PFP selected using the ISA procedure (red solid), as well as numerical values of the slopes ($S_{\mathrm{PFP-LM}}$) and 1σ uncertainties of a fit to these points. The GCMs shown in this figure required the largest positive offsets to the PSC formation temperature (see Methods), indicating the Arctic stratosphere for these eight models was the warmest of the 24 considered models, over the modern satellite era.

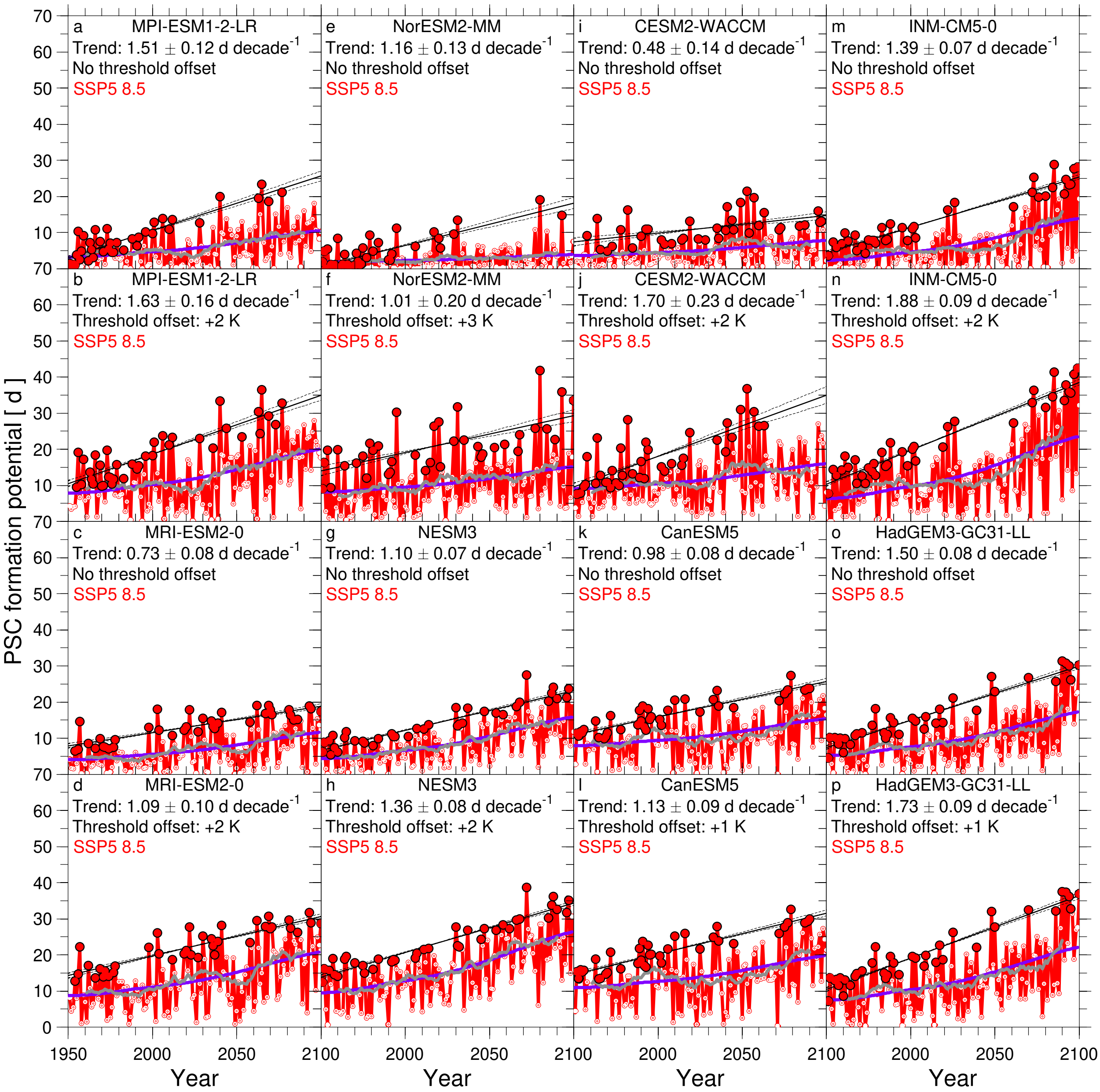

Supplementary Figure 4b. PSC Formation Potential (PFP) from the next eight CMIP6 GCMs for the SSP5-8.5 scenario, with and without adjustment of the temperature for formation of PSCs. Same as Supplementary Fig. 4a, for the next eight GCMs.

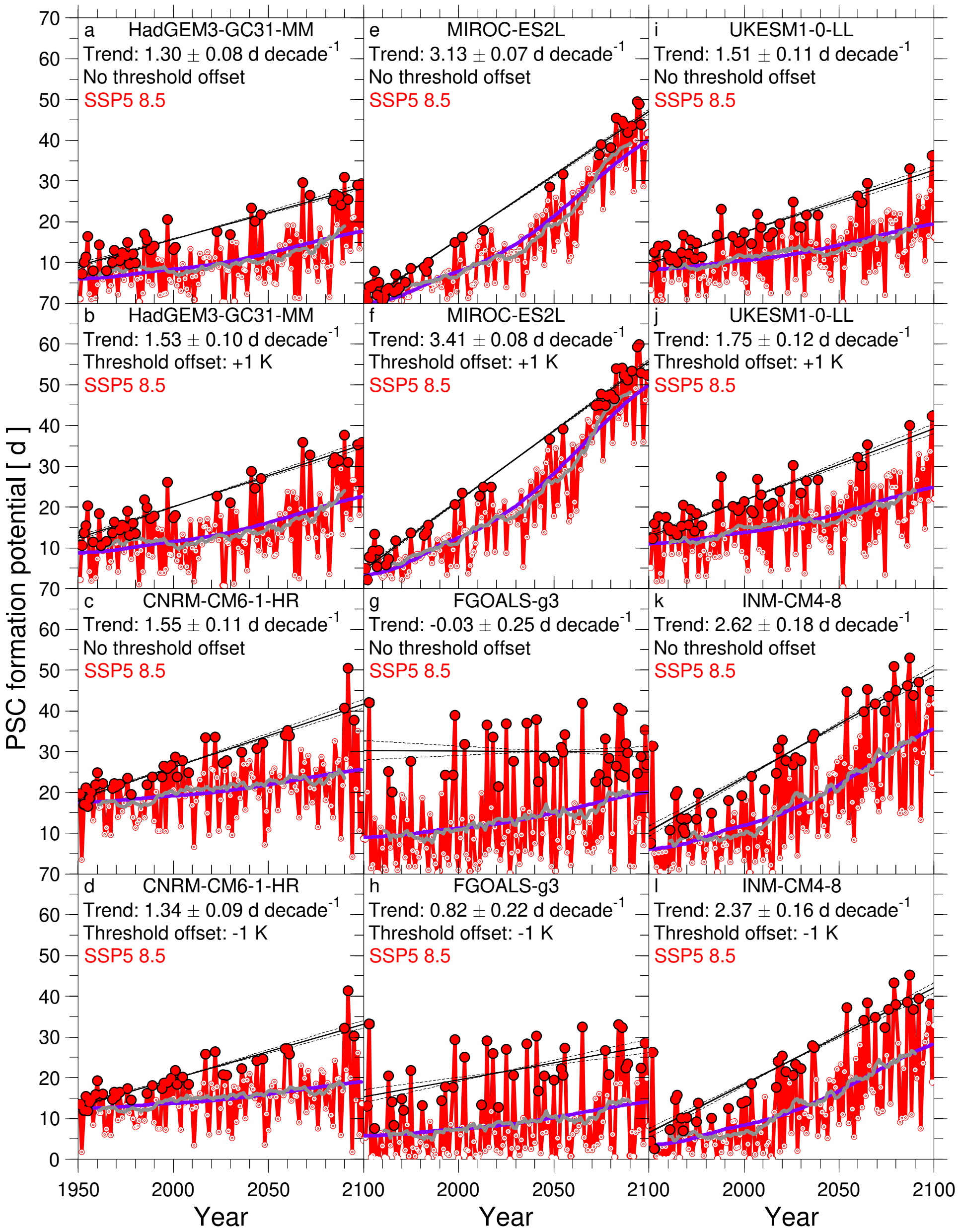

Supplementary Figure 4c. PSC Formation Potential (PFP) from the next six CMIP6 GCMs for the SSP5-8.5 scenario, with and without adjustment of the temperature for formation of PSCs. Same as Supplementary Fig. 4a, for the next six GCMs.

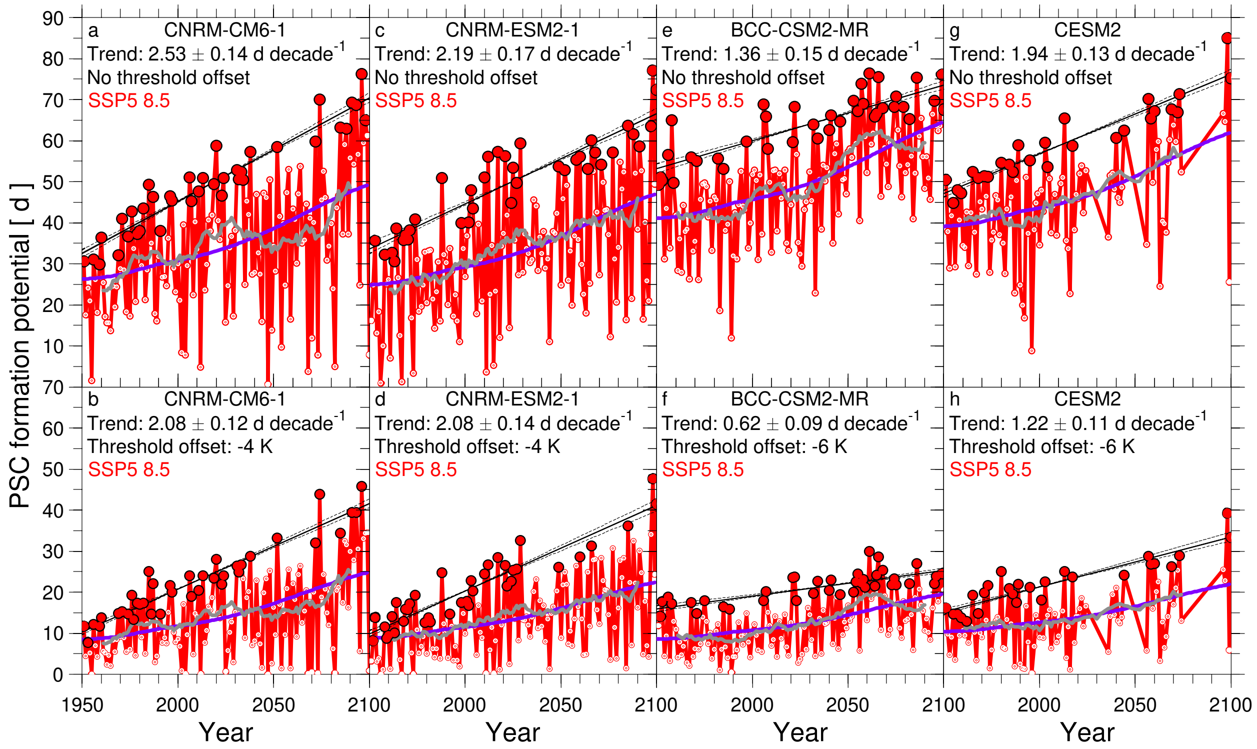

Supplementary Figure 4d. PSC Formation Potential (PFP) from the last four CMIP6 GCMs for the SSP5-8.5 scenario, with and without adjustment of the temperature for formation of PSCs. Same as Supplementary Fig. 4a, for the last four GCMs. The GCMs shown in this figure required largest negative offsets to the PSC formation temperature (see Methods), indicating the Arctic stratosphere for these GCMs was the coldest of the 26 considered models, over the modern satellite era.

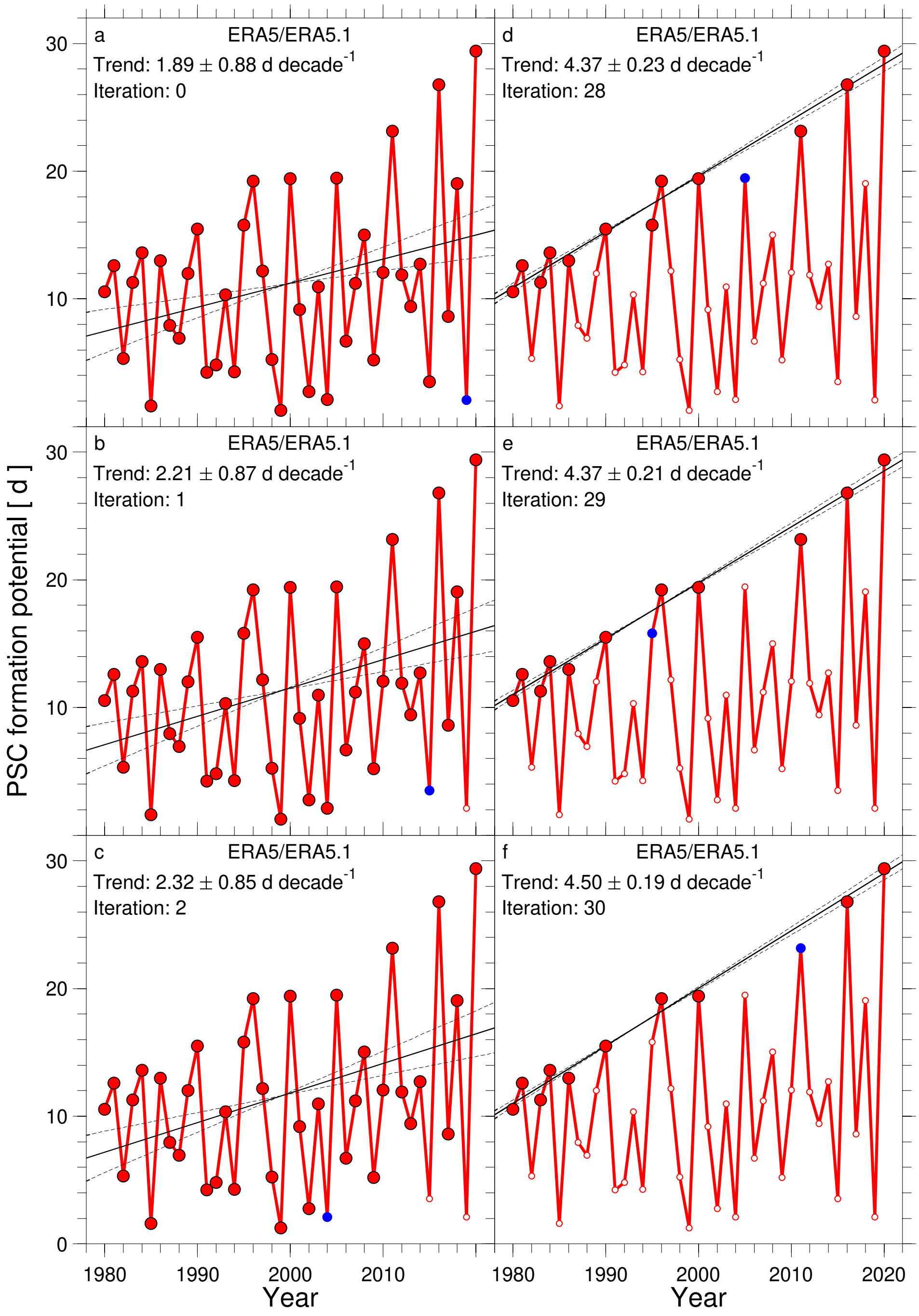

Supplementary Figure 5. Illustration of the ISA selection procedure. a-f, Each panel shows the same time series of PSC formation potential (PFP) from ERA5/ERA5.1. **a**, A least squares linear regression and uncertainty of the fit for all data and indication of PFP data point (blue) that lies furthest below the regression line; **b**, regression of remaining points (closed circles) and indication of the next point that lies furthest below the fit (blue); **c**, same as (b) for second iteration; **d**, same as (b), for iteration number 28; **e**, same as (b) for second to last iteration; **f**, fit to final selection of local maxima of PFP (PFP^{LM}), constituted by the upper quartile (25%) of points relative to the various regression lines.

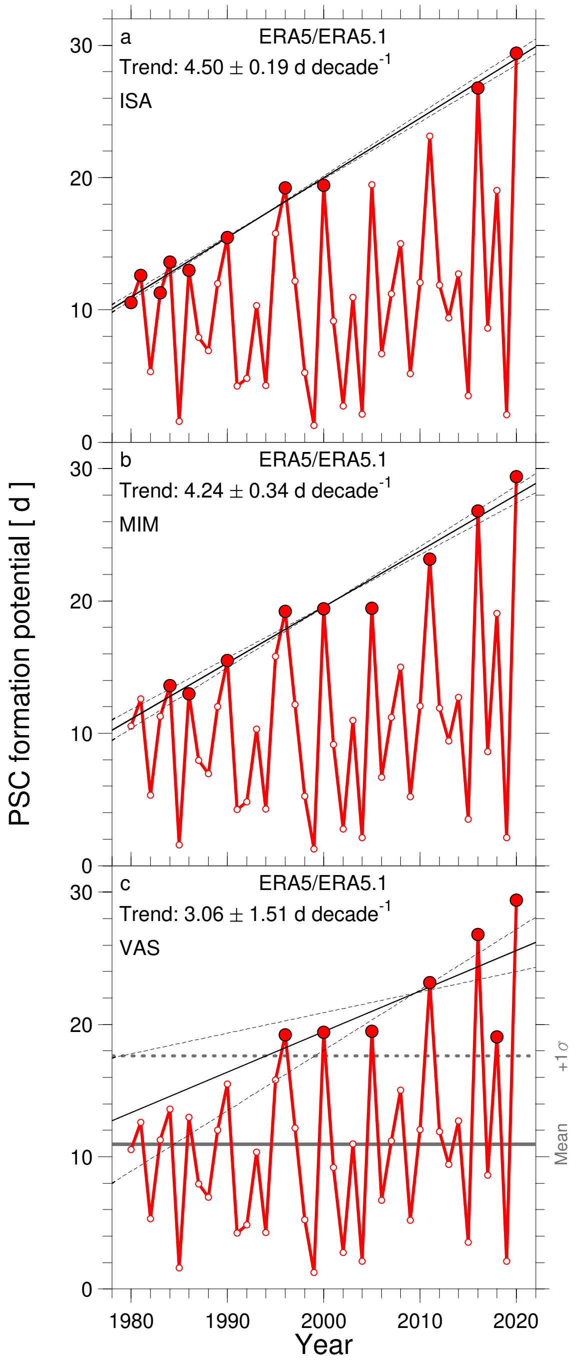

Supplementary Figure 6. Comparison of various analysis procedures for trends in maxima of PSC formation potential (PFP). a-c, Each panel shows the same time series of PFP from ERA5/ERA5.1, along with a linear, least squares fit (solid line) and 1σ uncertainty of the fit (dashed lines) to the solid red circles shown in each panel. **a**, selection of local maxima of PFP by the Iterative Selection Approach (ISA); **b**, selection of local maxima of PFP by the Maximum in the Interval Method (MIM) for 5 year intervals; **c**, selection of PFP maxima by the Value Above Sigma (VAS) approach. For panel c, the mean and mean plus one standard deviation values of PFP are indicated.

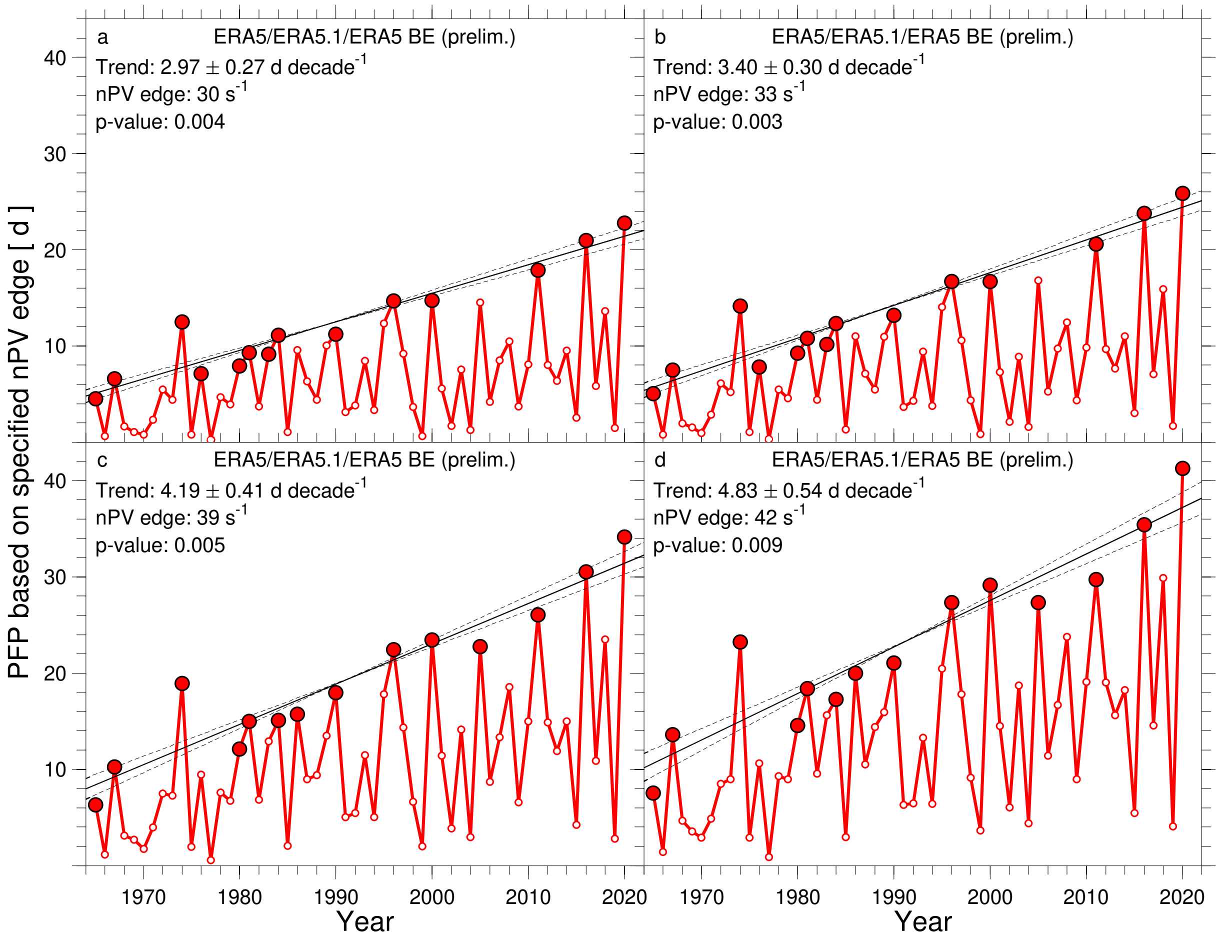

Supplementary Figure 7. PSC Formation Potential (PFP) as a function of time, ERA5/ERA5.1 combined with the ERA5 back extension (BE) (preliminary version). Same as Fig. 3b, except for the use of values of nPV for the horizontal boundary of the vortex of 30 sec^{-1} (**a**), 33 sec^{-1} (**b**), 39 sec^{-1} (**c**), and 42 sec^{-1} (**d**).

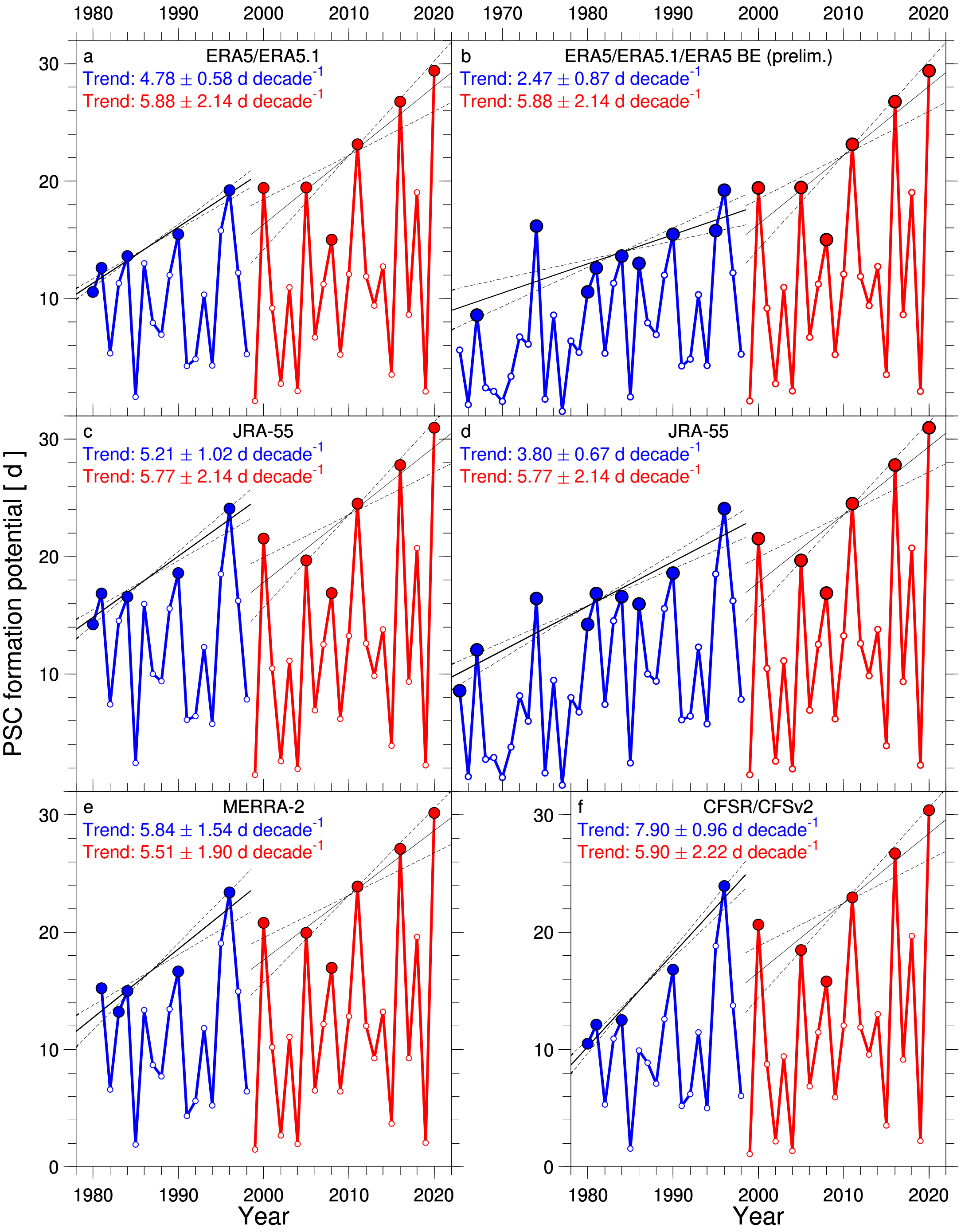

Supplementary Figure 8. Time series of PSC Formation Potential (PFP) from ERA5/ERA5.1 (ref.[1]) for the SSU/TOVS era (blue) and the AMSU/ATOVS era (red), the data points selected by ISA that constitute PFP^{LM} (solid circles), as well as the numerical values of the slopes and uncertainties in PFP^{LM} for the respective eras.

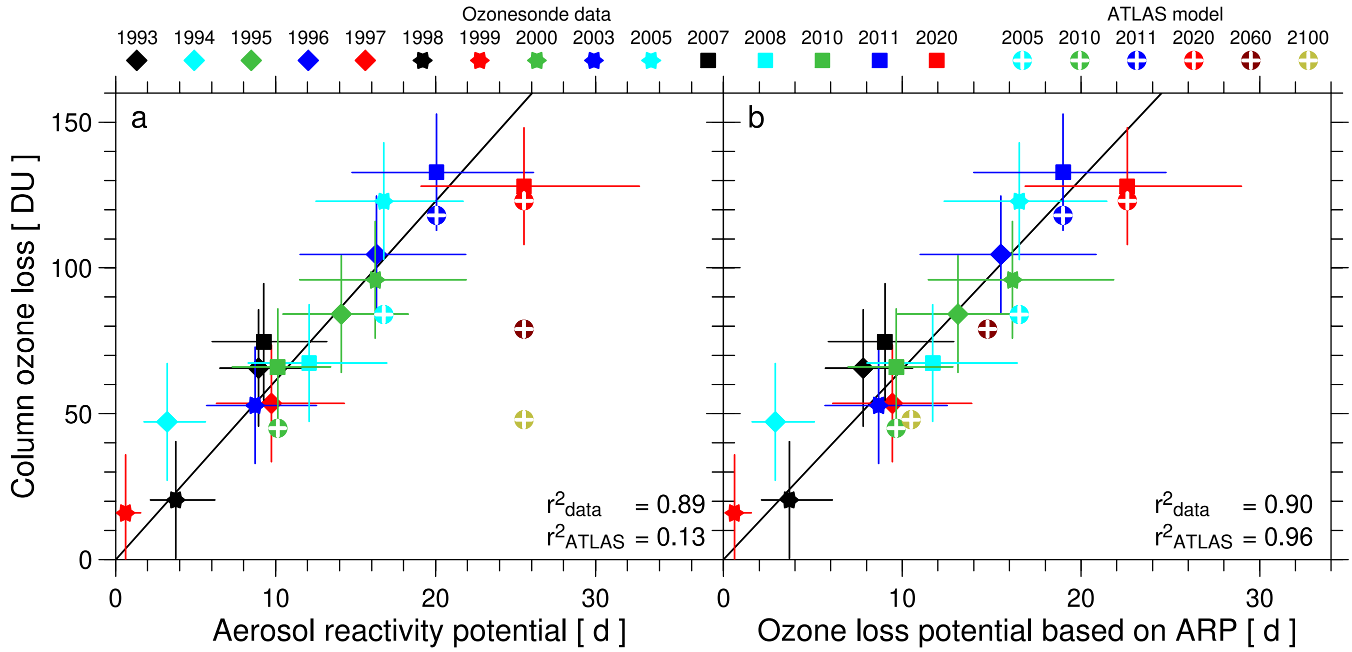

Supplementary Figure 9. Chemical loss of Arctic ozone. Same as Fig. 1 of the main article, except the chemical ozone loss of ozone is displayed as a function of Aerosol Reactivity Potential (ARP) rather than PSC Formation Potential (PFP).

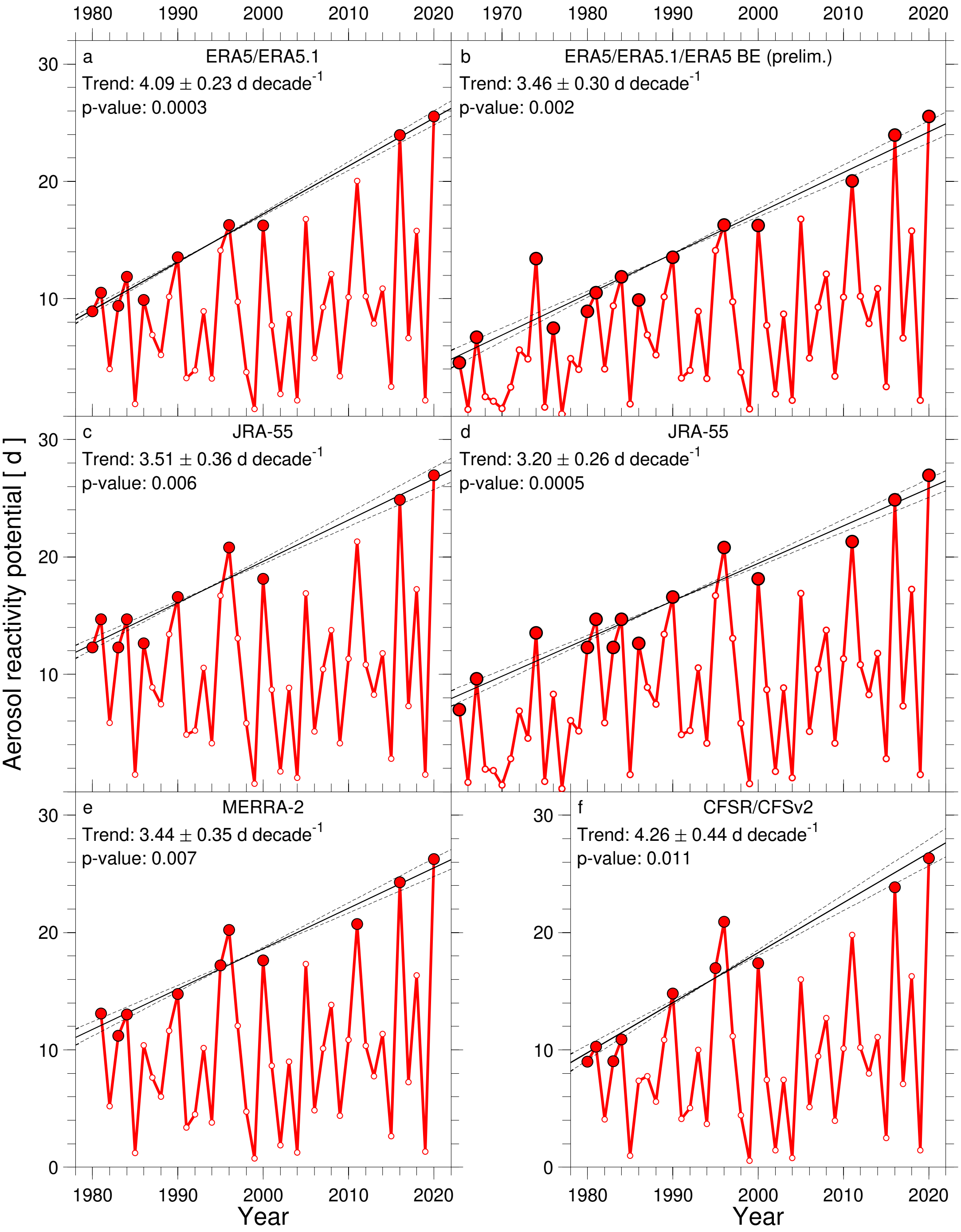

Supplementary Figure 10. Aerosol Reactivity Potential (ARP) as a function of time. Same as Fig. 3 of the main article, except for ARP rather than PSC Formation Potential (PFP). A linear, least squares fit (solid line) and 1 σ uncertainty (dashed lines) to the solid red circles are shown in each panel, along with numerical values of the slopes for the local maxima of ARP ($S_{\mathrm{ARP-LM}}$) found using ISA, the 1σ uncertainties of these slopes ($\Delta S_{\mathrm{ARP-LM}}$), as well as p-values for the quantity $S_{\mathrm{ARP-LM}}/\Delta S_{\mathrm{ARP-LM}}$

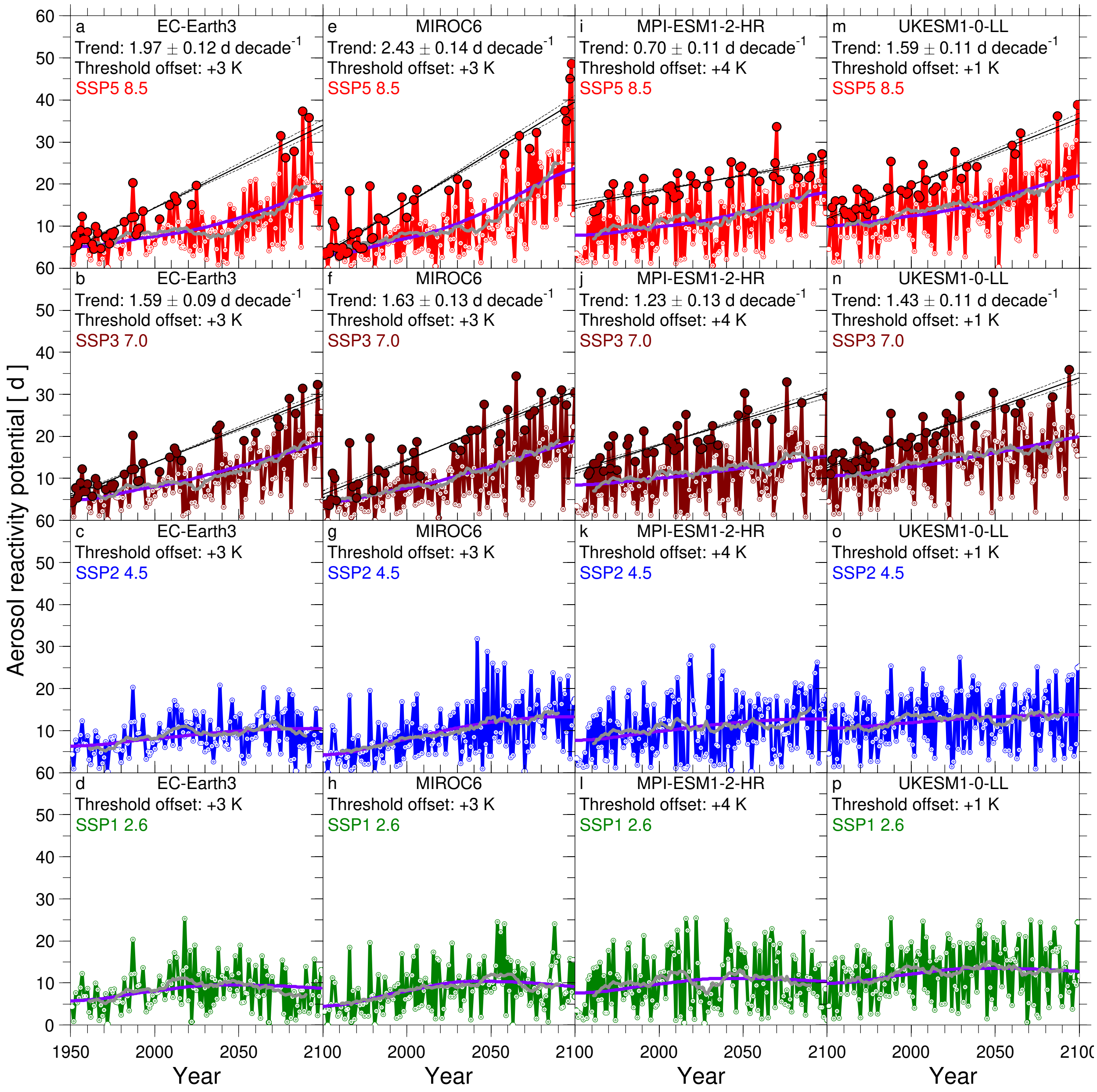

Supplementary Figure 11. Aerosol Reactivity Potential (ARP), 1950 to 2100, for time invariant H_2O from GCMs for various SSP scenarios. Same as Fig. 5 of the main article, except for ARP rather than PSC Formation Potential (PFP).

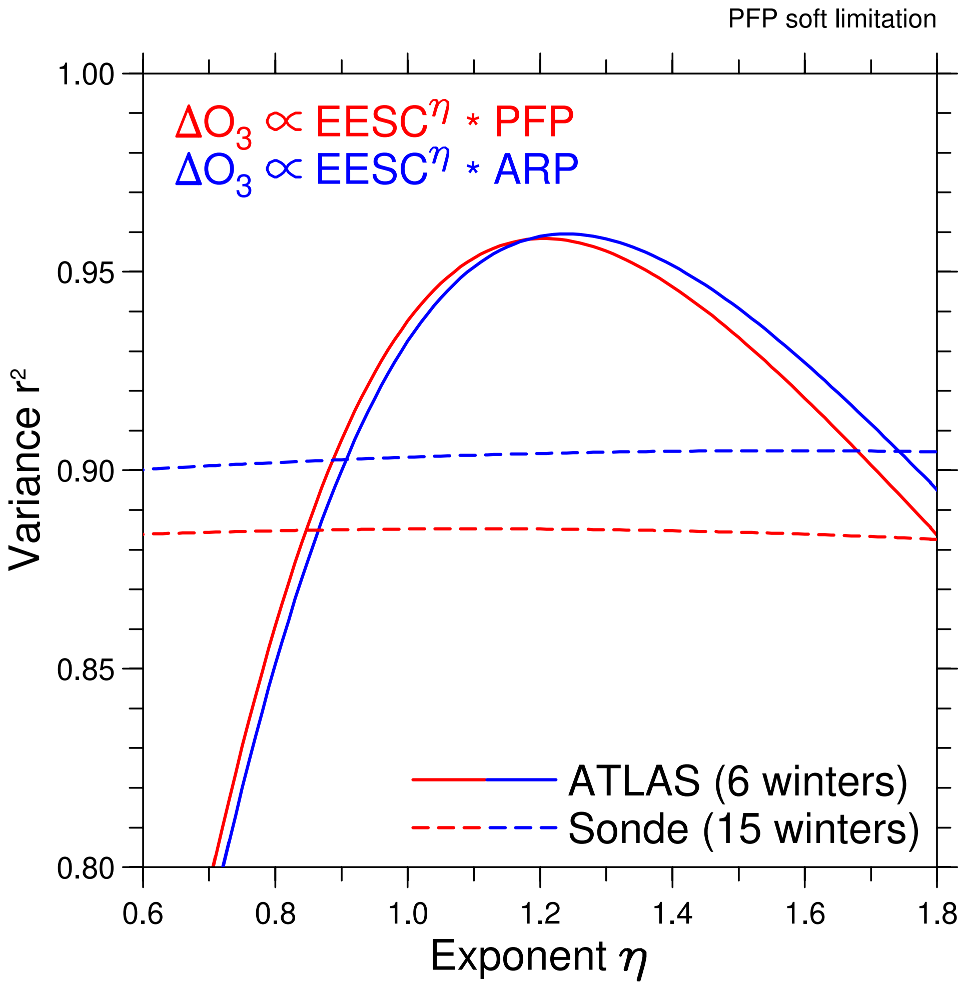

Supplementary Figure 12. Variance as a function of exponent. The variance in observed (Sonde) and modeled (ATLAS) ΔO_3 (shown in the ordinate of Fig. 1) that is explained by the quantity OLP (Ozone Loss Potential) that is computed using PFP (PSC Formation Potential), as a function of the value of the exponent η in the expression for OLP (Eq. 7) (red). Here, variance is reported as the square of the correlation coefficient between ΔO_3 and the value of OLP associated with the specific 6 or 15 winters for which ΔO_3 has either been modeled or observed, respectively. The results shown in blue are for the use of ARP (Aerosol Reactivity Potential), rather than PFP, in the expression for OLP (Eq. 8).

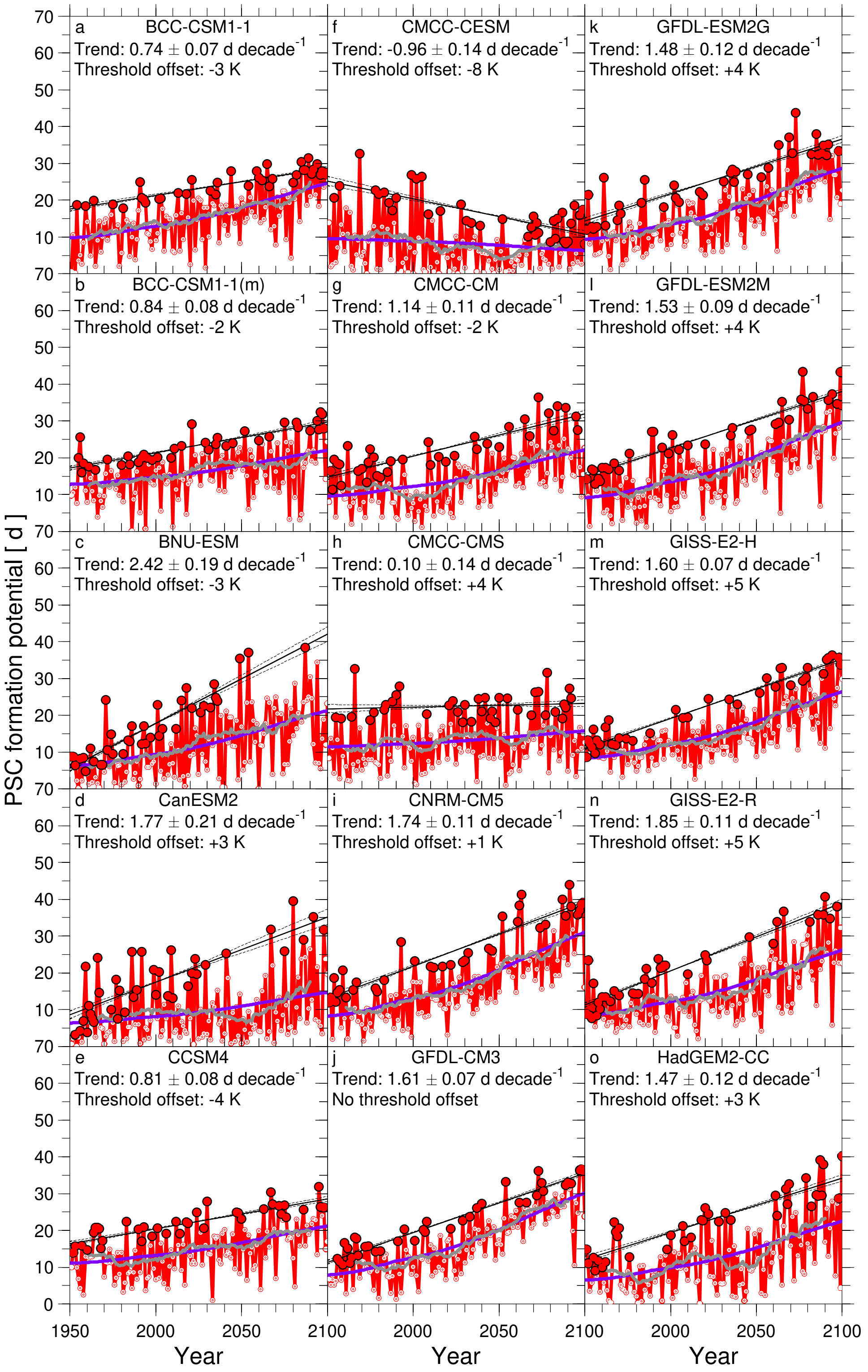

Supplementary Figure 13a. PSC Formation Potential (PFP) from 1950 to 2100 from first 15 CMIP5 GCMs for the RCP 8.5 scenario. Same as Fig. 4 of the main article, except results are shown for 15 CMIP5 GCMs.

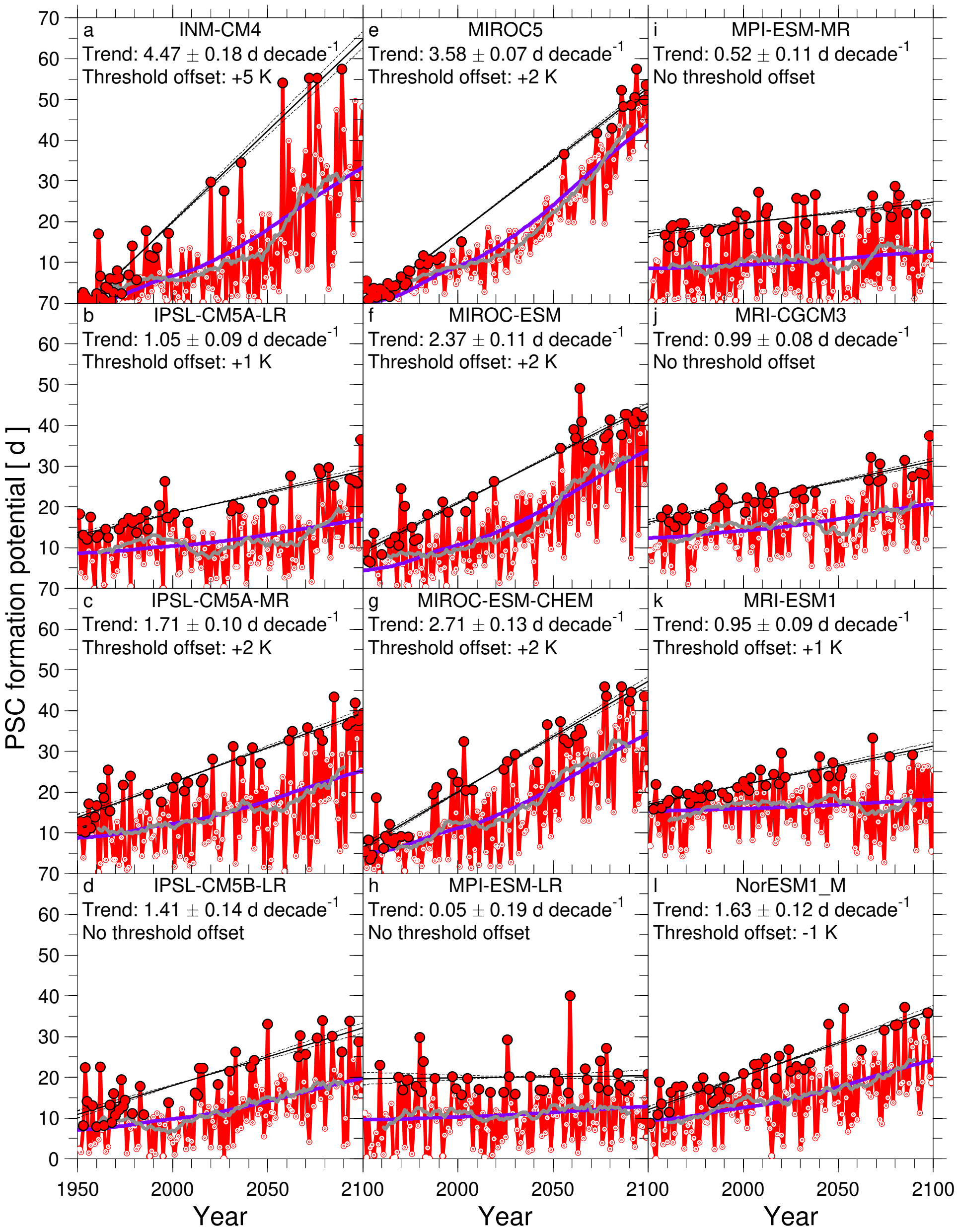

Supplementary Figure 13b. PSC Formation Potential (PFP) from 1950 to 2100 from last 12 CMIP5 GCMs for the RCP 8.5 scenario. Same as Fig. 4 of the main article, except results are shown for 12 CMIP5 GCMs.

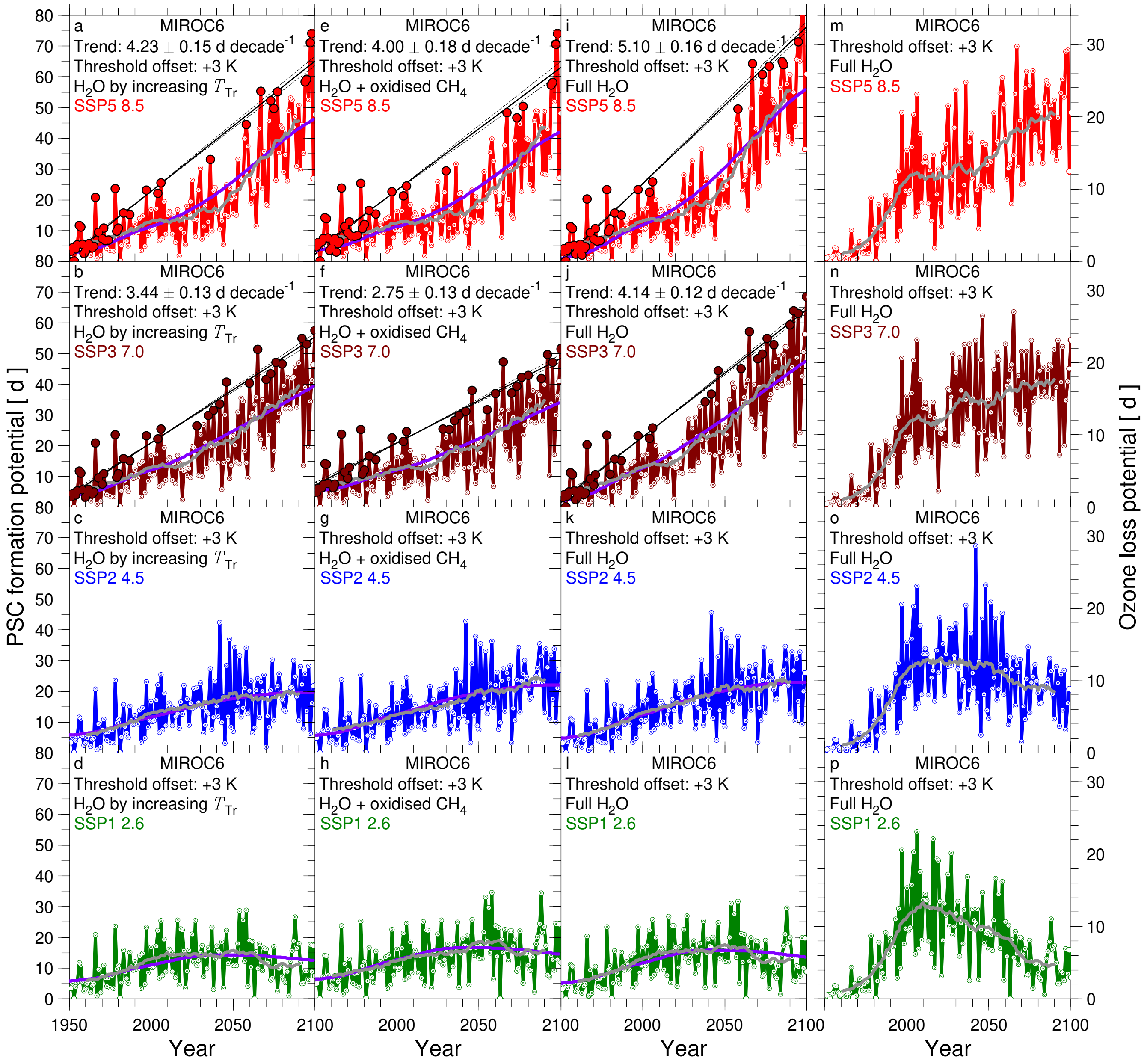

Supplementary Figure 14a. PSC Formation Potential (PFP) and Ozone Loss Potential ($EESC^{1.2}$×PFP), 1950 to 2100, from MIROC6 for variable H_2O, various SSP scenarios. Same as Fig. 7 of the main article, except for the MIROC6 CMIP6 GCM.

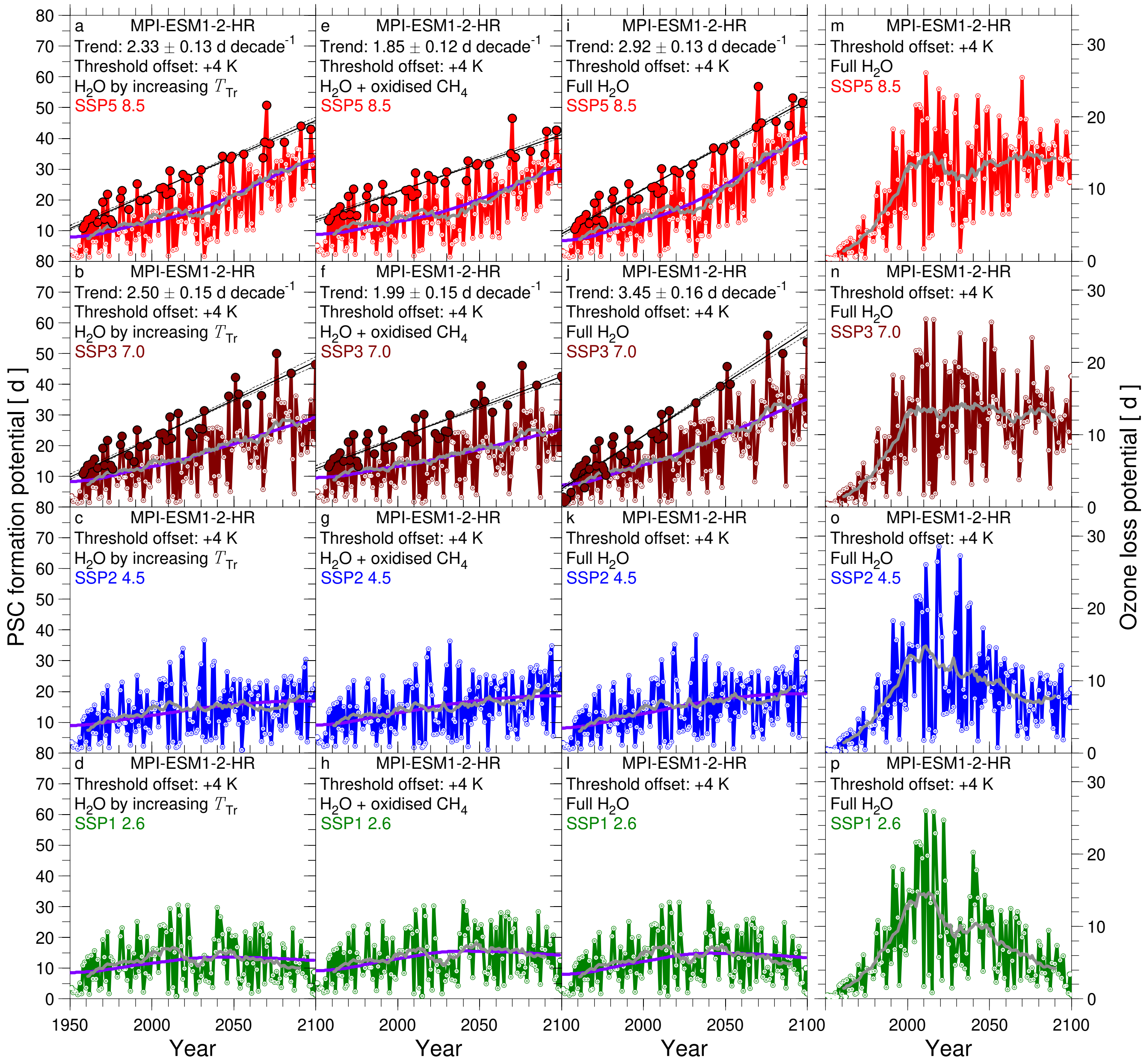

Supplementary Figure 14b. Same as Fig. 7 of the main article, except for the MPI-ESM1-2-HR CMIP6 GCM.

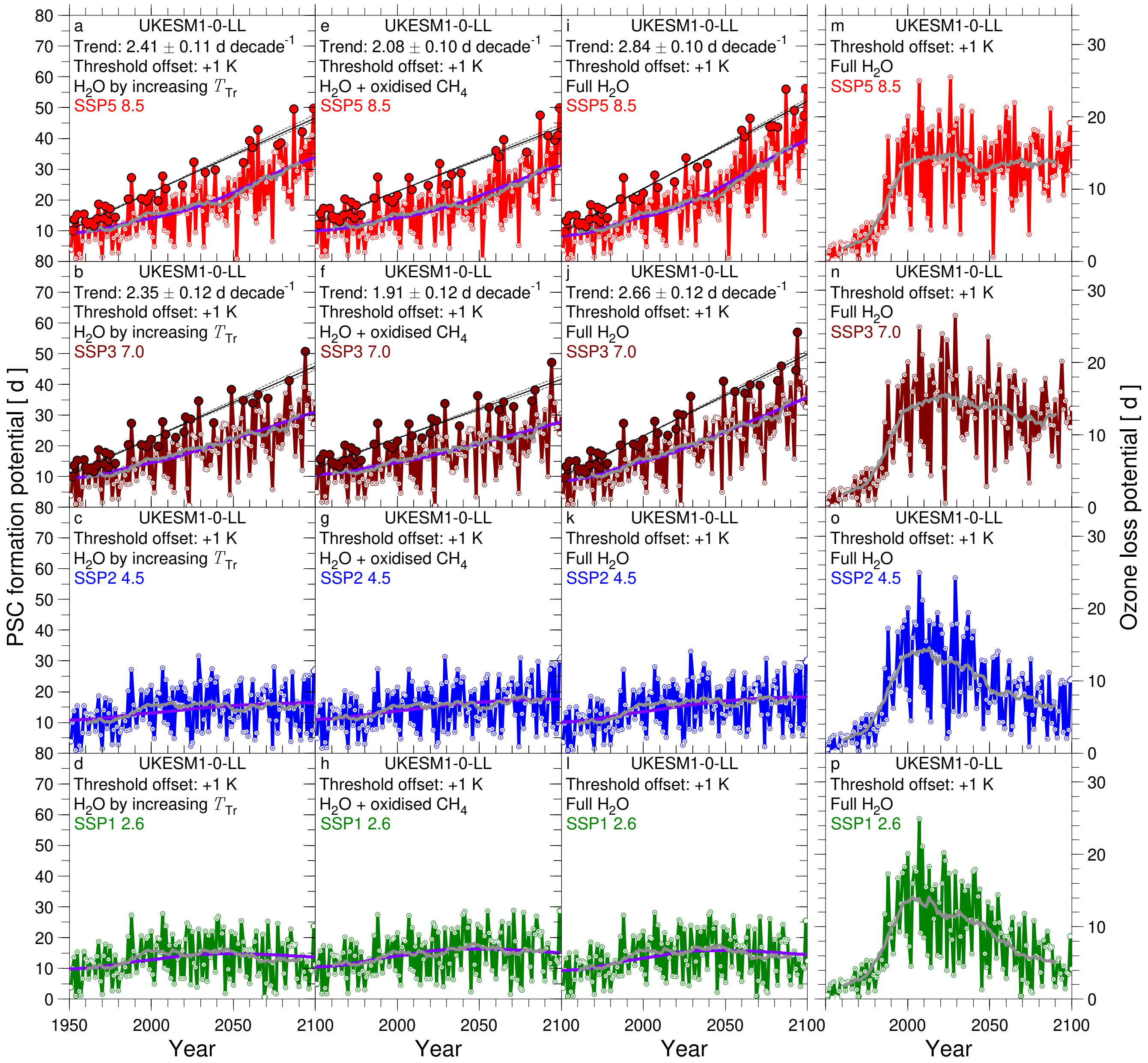

Supplementary Figure 14c. Same as Fig. 7 of the main article, except for the UKESM1-0-LL CMIP6 GCM.

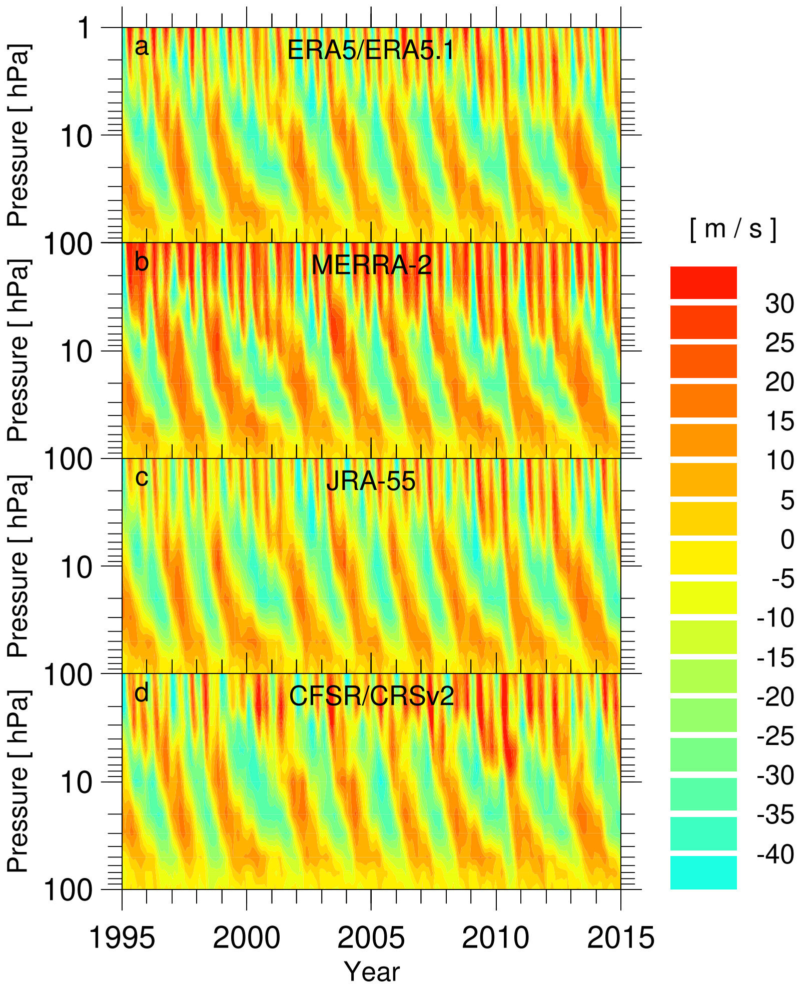

Supplementary Figure 15. Observed Quasi-Biennial Oscillation (QBO) of equatorial, zonal winds from four data centres. a-d, Zonal, monthly mean speed of the equatorial wind from ERA5/ERA5.1, MERRA-2, JRA-55, and CFSR/CRSv2. Positive values denote westerly winds and negative values denote easterly winds.

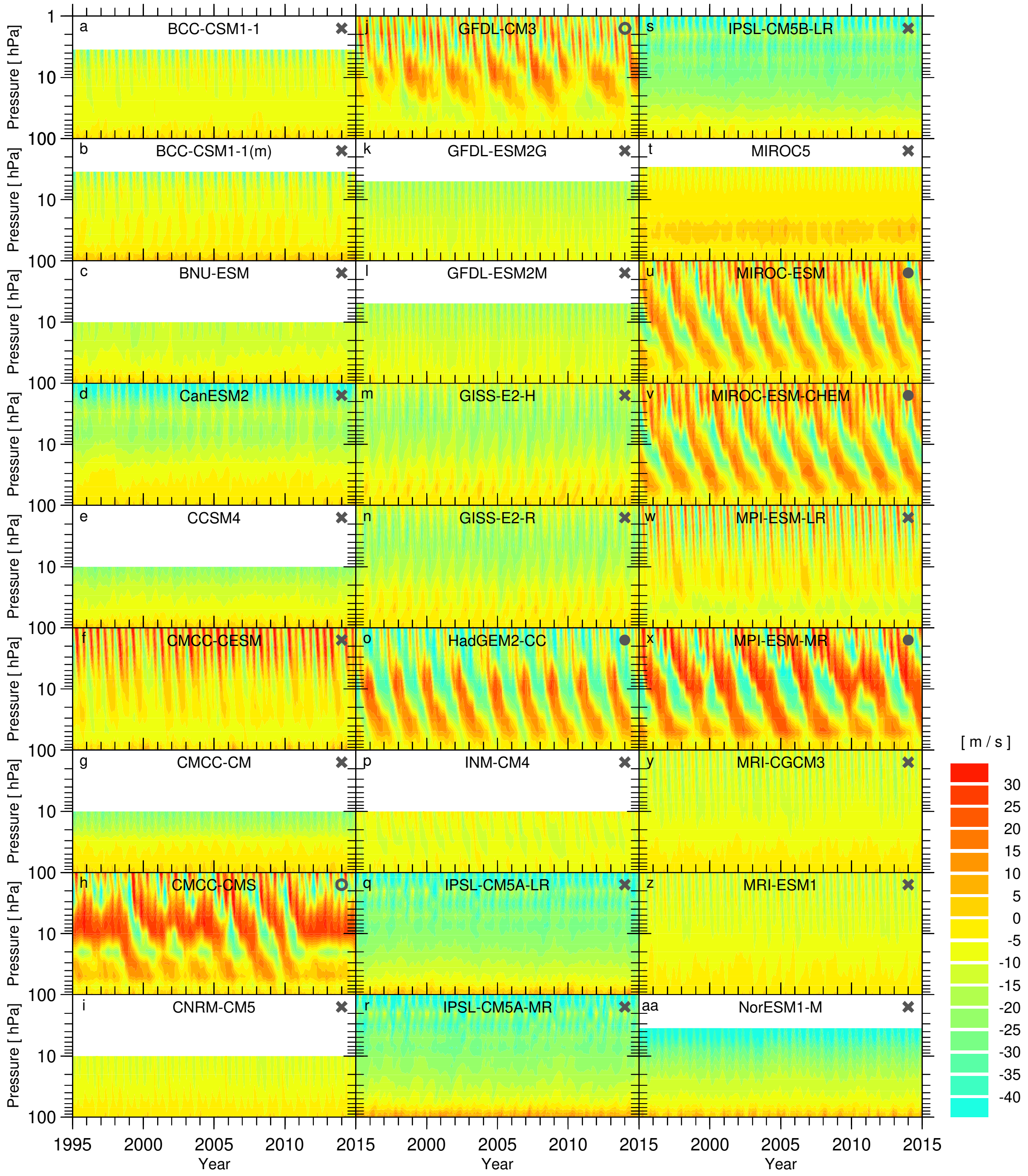

Supplementary Figure 16a. Computed Quasi-Biennial Oscillation (QBO) of equatorial, zonal winds from CMIP5 GCMs. a-aa, Zonal, monthly mean speed of the equatorial wind from the 27 CMIP5 GCM simulations used in the study, for the same time period used in Supplementary Fig. 15. Positive values denote westerly winds and negative values denote easterly winds. The symbols ✖, ○, and ● on each panel denote either no apparent QBO, perhaps a QBO, and the presence of a QBO that bears a strong resemblance to the observed QBO, respectively.

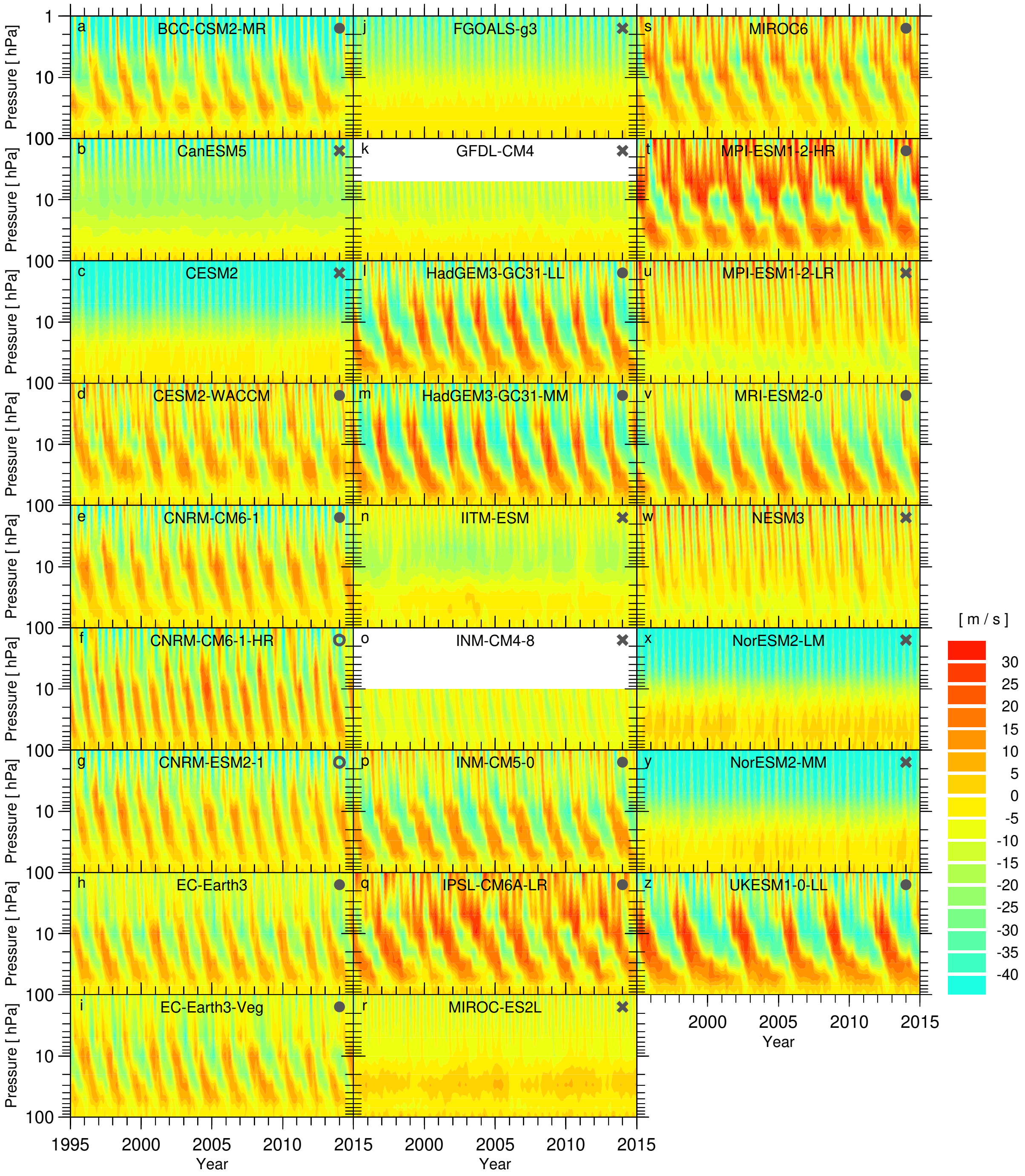

Supplementary Figure 16b. Computed Quasi-Biennial Oscillation (QBO) of equatorial, zonal winds from CMIP6 GCMs. a-z, Zonal, monthly mean speed of the equatorial wind from the 26 CMIP6 GCM simulations used in the study, for the same time period used in Supplementary Fig. 15. Positive values denote westerly winds and negative values denote easterly winds. The symbols ✖, ○, and ● on each panel denote either no apparent QBO, perhaps a QBO, and the presence of a QBO that bears a strong resemblance to the observed QBO, respectively.

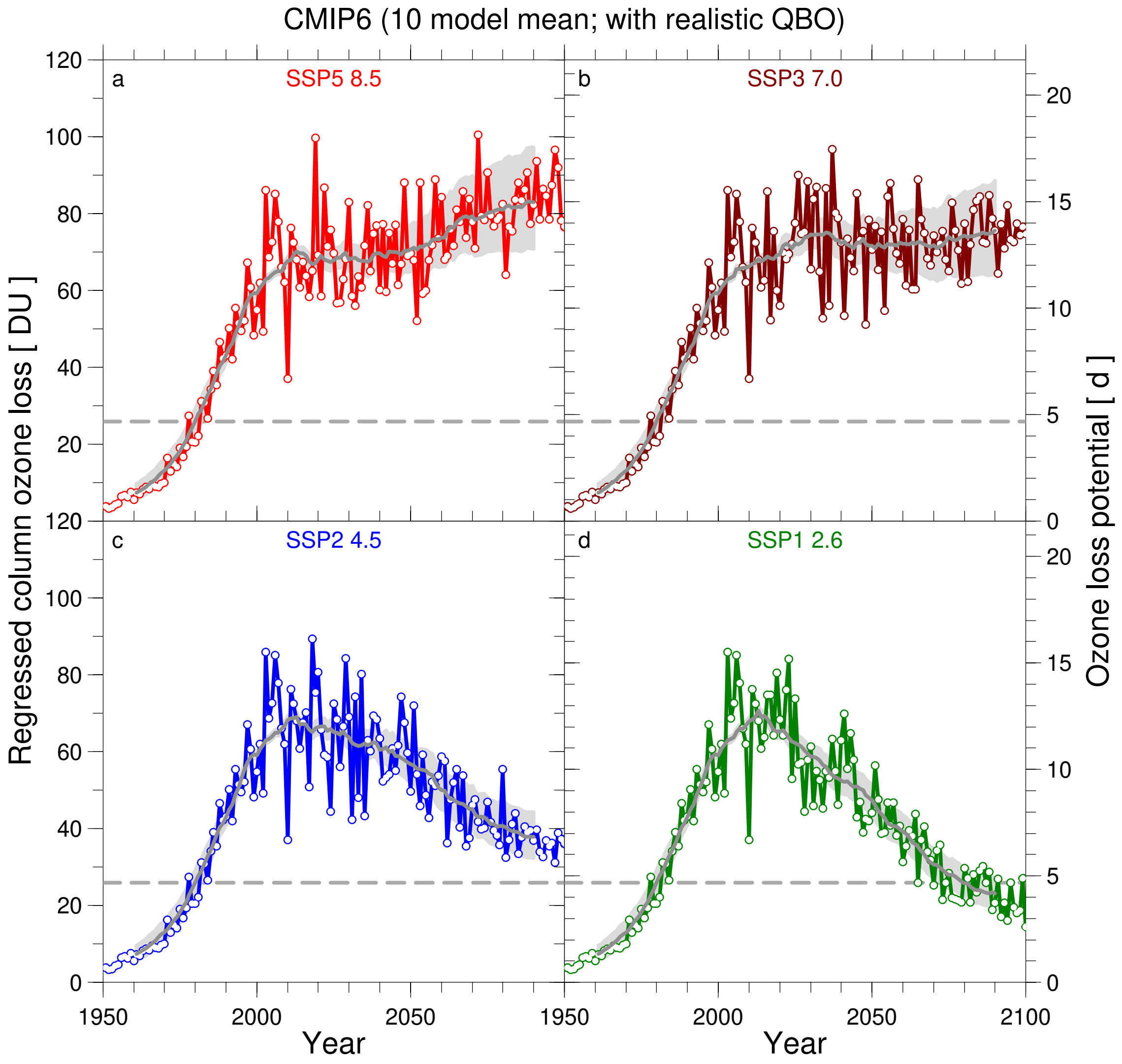

Supplementary Figure 17a. Ensemble mean regressed column ozone loss and Ozone Loss Potential (OLP), variable H_2O, for CMIP6 models with realistic Quasi-Biennial Oscillation (QBO). Same as Fig. 9 of the main article, except the ensemble mean is for the subset of 10 CMIP6 GCMs of the 20 GCMs that submitted results for all four SSP scenarios that exhibit a realistic QBO (CESM2-WACCM, CNRM-CM6-1, EC-Earth3, EC-Earth3-Veg, INM-CM5-0, IPSL-CM6A-LR, MIROC6, MPI-ESM1-2-HR, MRI-ESM2-0, UKESM1-0-LL), as denoted by the symbol ● in Supplementary Fig. 16b.

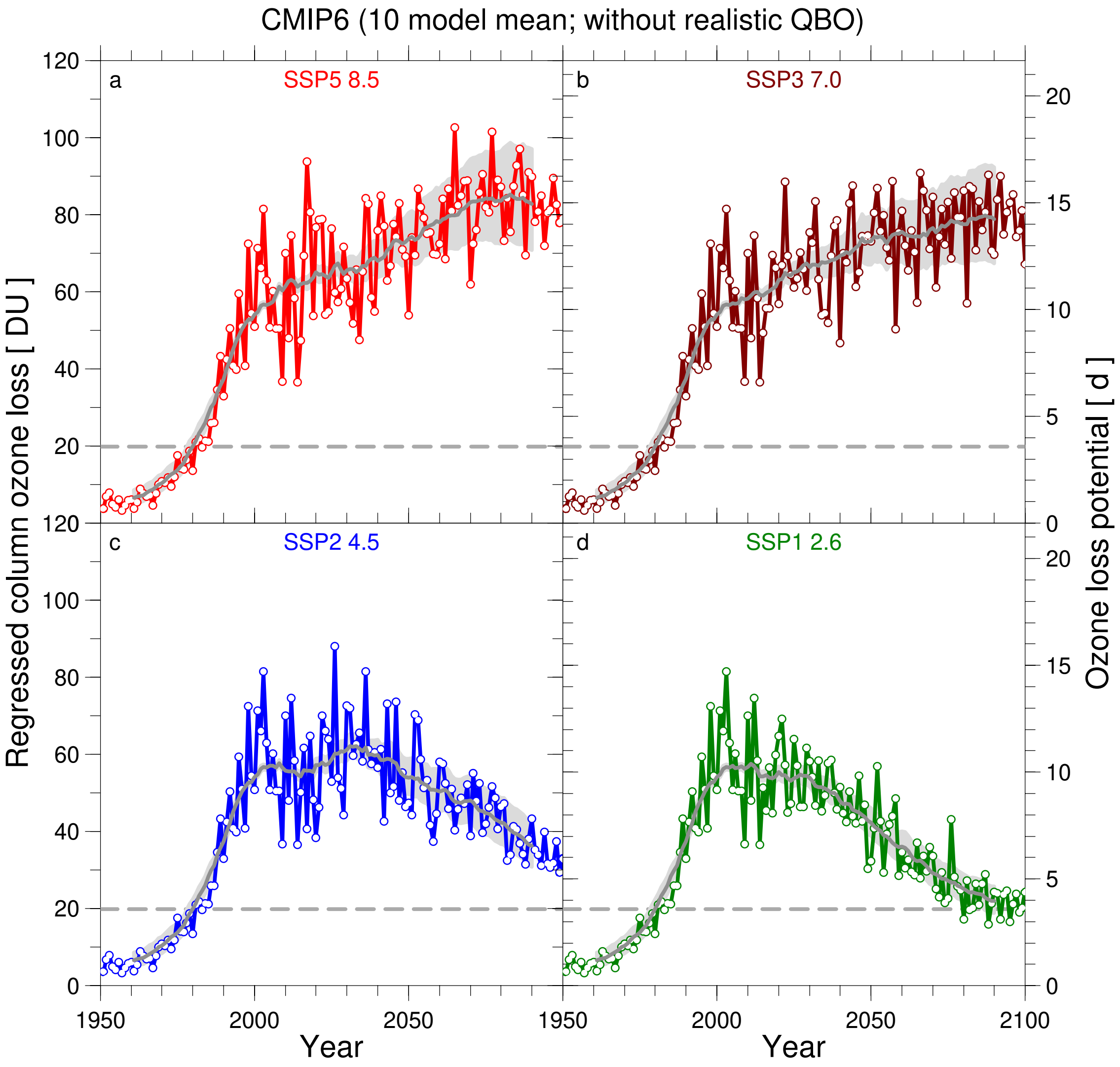

Supplementary Figure 17b. Ensemble mean regressed column ozone loss and Ozone Loss Potential (OLP), variable H_2O, for CMIP6 models without a realistic Quasi-Biennial Oscillation (QBO). Same as Fig. 9 of the main article, except the ensemble mean is for the subset of 10 CMIP6 GCMs of the 20 GCMs that submitted results for all four SSP scenarios that do not exhibit a realistic QBO (CanESM5, CNRM-CM6-1-HR, CNRM-ESM2-1, FGOALS-g3, IITM-ESM, INM-CM4-8, MIROC-ES2L, MPI-ESM1-2-LR, NorESM2-LM, NorESM2-MM), as denoted by the symbols ✖ and ○ in Supplementary Fig. 16b.

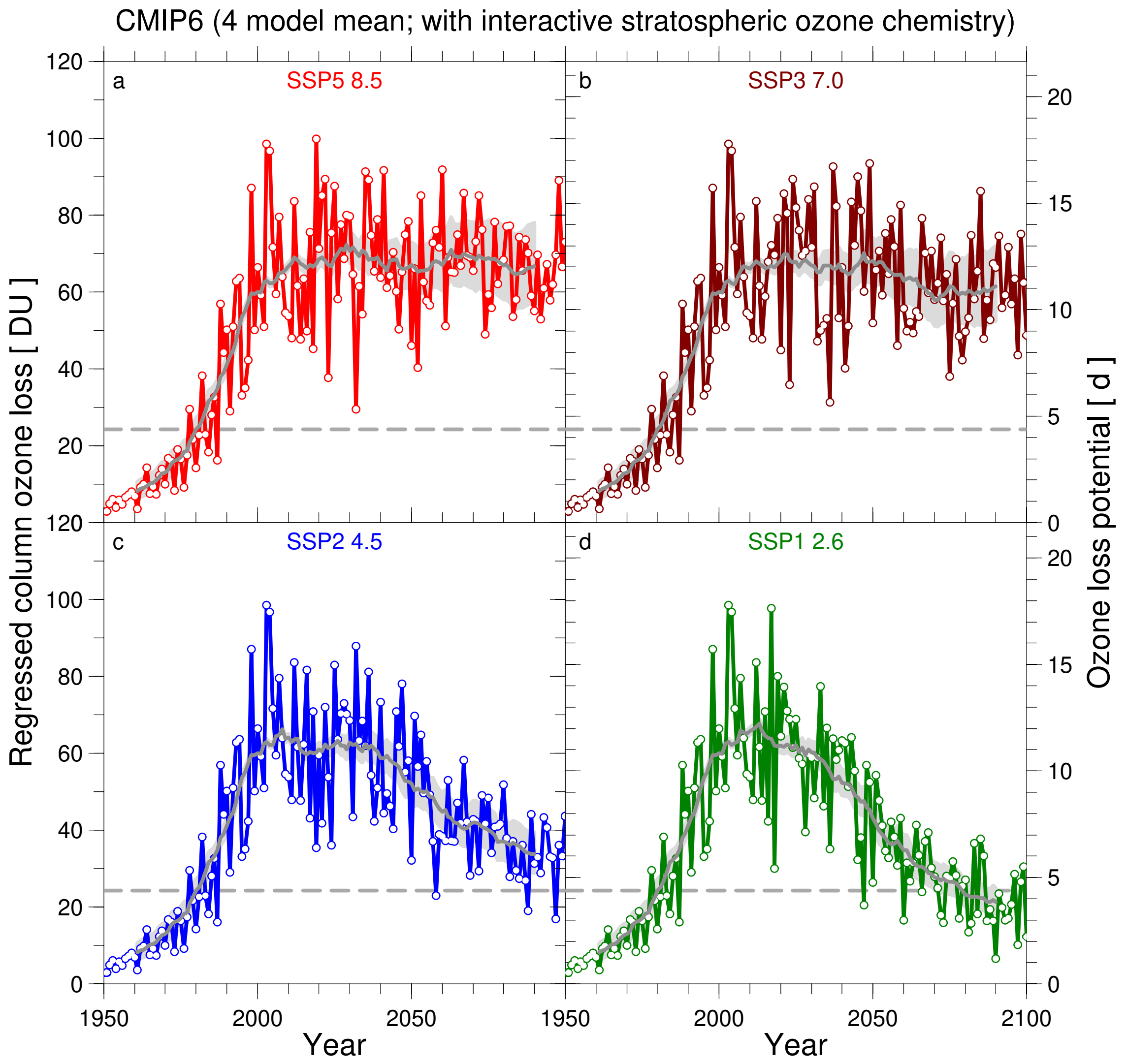

Supplementary Figure 18. Ensemble mean regressed column ozone loss and Ozone Loss Potential (OLP), variable H_2O, for CMIP6 GCMs with interactive chemistry. Same as Fig. 9 of the main article, except the ensemble mean is for the subset of four CMIP6 GCMs (CESM2-WACCM, CNRM-ESM2-1, MRI-ESM2-0, UKESM1-0-LL) that have fully interactive stratospheric chemistry.

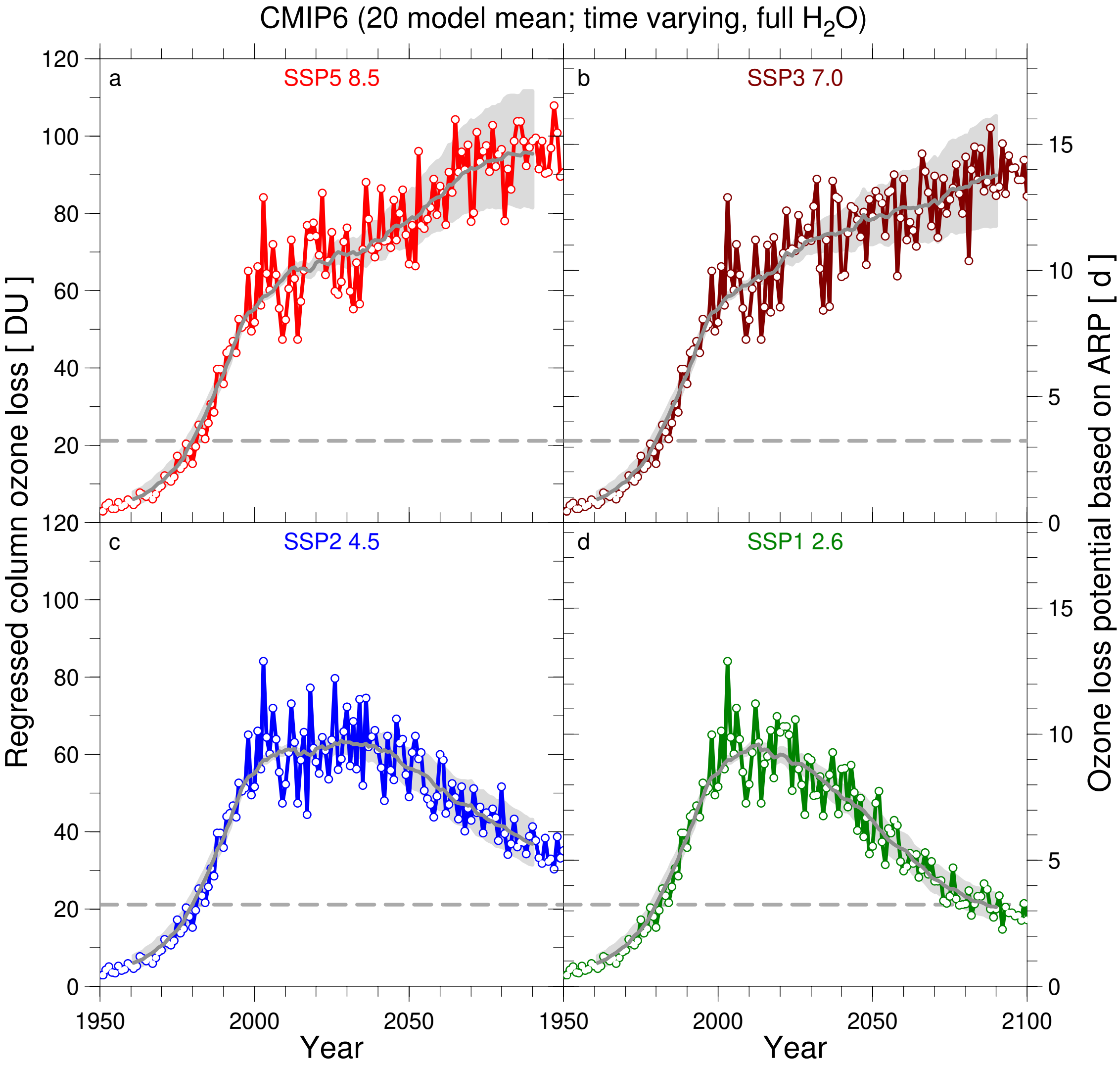

Supplementary Figure 19. Ensemble mean regressed column ozone loss and Ozone Loss Potential (OLP) based on Aerosol Reactivity Potential (ARP), variable H_2O, for CMIP6 GCMs. Same as Fig. 9 of the main article, except the ARP is used, rather than PSC Formation Potential (PFP), for the computations of OLP and ΔO_3^{REG}.

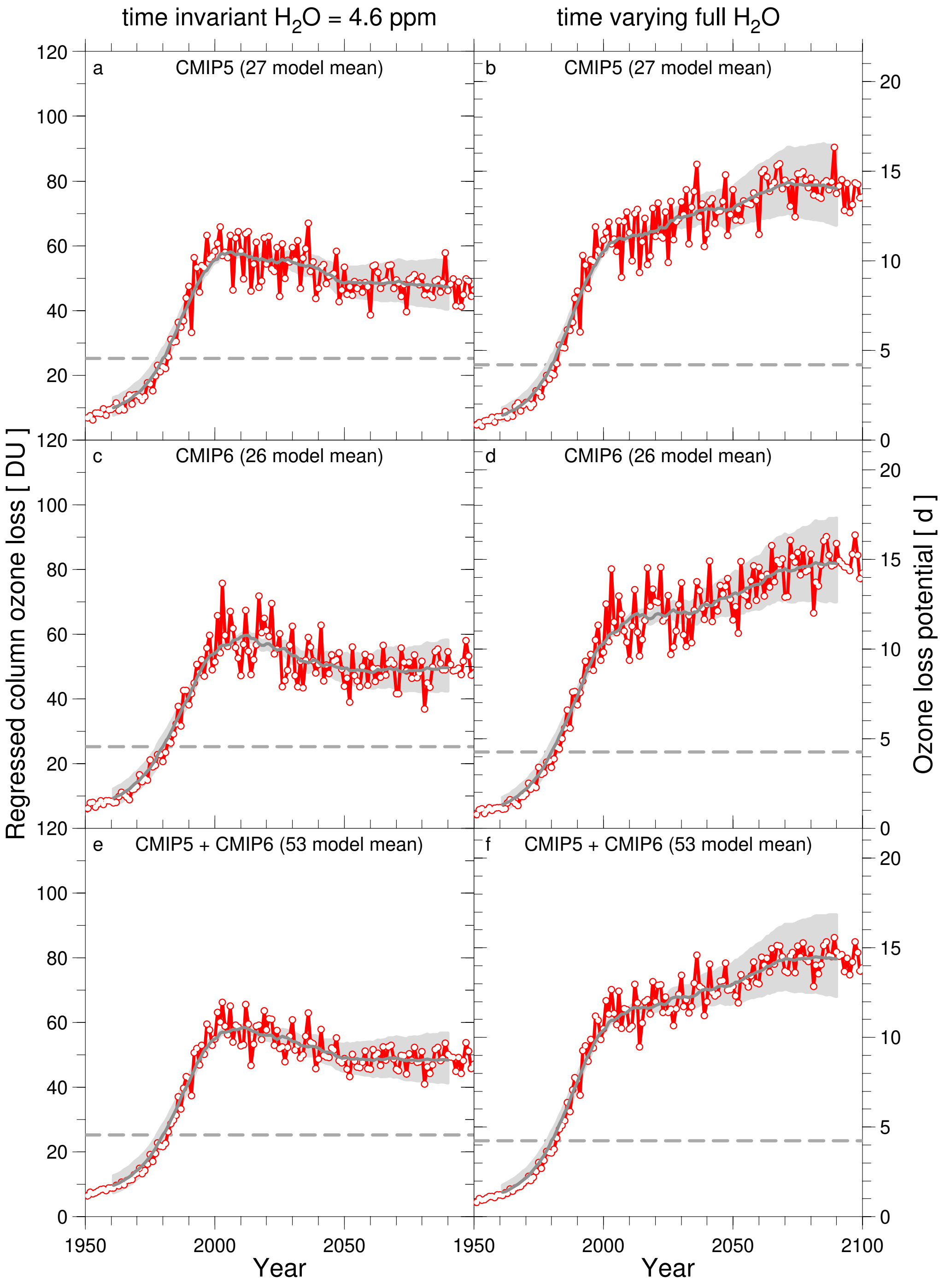

Supplementary Figure 20. Ensemble mean regressed column ozone loss and Ozone Loss Potential (OLP), constant and variable H_2O, for CMIP5 and CMIP6 models with 8.5 W m^{-2} radiative forcing of climate. Same as Fig. 8a (constant H_2O) and Fig. 9a (time varying H_2O) of the main article, for the 27 CMIP5 GCMs that have archived stratospheric winds and temperature for RCP 8.5 (panels **a** and **b**), the 26 CMIP6 GCMs that have archived results for SSP5-8.5 (panels **c** and **d**), and the multi-model ensemble mean for both RCP 8.5 and SSP5-8.5 runs of CMIP5 and CMIP6 GCMs, respectively, for constant H_2O (panel **e**) and the full effect of rising stratospheric H_2O (i.e., CH_4 oxidation and thermodynamics) (panel **f**).

Supplementary Table 1 Description of GCMs.

Details of the CMIP5 and CMIP6 GCMs used in this study.

Modeling center (or Group)	Institute ID	Model(s)	Reference(s)
Beijing Climate Center, China Meteorological Administration	BCC	BCC-CSM1-1, BCC-CSM1-1(m)	Wu et al. (2014)[3]
		BCC-CSM2-MR	Wu et al. (2019)[4]
College of Global Change and Earth System Science, Beijing Normal University	GCESS	BNU-ESM	Ji et al. (2014)[5]
Canadian Centre for Climate Modelling and Analysis	CCCMA	CanESM2	
		CanESM5	Swart et al. (2019)[6]
National Center for Atmospheric Research	NCAR	CCSM4<br>CESM2,<br>CESM2-WACCM	Gent et al. (2011)[7]<br>Danabasoglu et al. (2020)[8]
Centro Euro-Mediterraneo per I Cambiamenti Climatici	CMCC	CMCC-CESM<br>CMCC-CM,<br>CMCC-CMS	Fogli et al. (2011)[9]; Vichi et al. (2011)[10]<br>Scoccimarro et al. (2011)[11]
Centre National de Recherches Météorologiques / Centre Européen de Recherche et Formation Avancée en Calcul Scientifique	CNRM-CERFACS	CNRM-CM5	Voldoire et al. (2013)[12]
		CNRM-CM6-1,<br>CNRM-CM6-1-HR,<br>CNRM-ESM2-1	Voldoire et al. (2019)[13]
EC-Earth consortium	EC-Earth	EC-Earth3,<br>EC-Earth3-Veg	EC-Earth Consortium (2019a)[14]<br>EC-Earth Consortium (2019b)[15]
Chinese Academy of Sciences	CAS	FGOALS-g3	Li (2019)[16]
NOAA Geophysical Fluid Dynamics Laboratory	NOAA GFDL	GFDL-CM3<br>GFDL-ESM2G,<br>GFDL-ESM2M	Donner et al. (2011)[17]<br>Dunne et al. (2012, 2013)[18,19]
		GFDL-CM4	Held et al. (2019)[20]
NASA Goddard Institute for Space Studies	NASA GISS	GISS-E2-H,<br>GISS-E2-R	Schmidt et al. (2014)[21]
Met Office Hadley Centre	MOHC	HadGEM2-CC	Martin et al. (2011)[22]; Collins et al. (2011)[23]
		HadGEM3-GC31-LL,<br>HadGEM3-GC31-MM,<br>UKESM1-0-LL	Andrews et al. (2019)[24]
Centre for Climate Change Research, Indian Institute of Tropical Meteorology Pune	CCCR-IITM	IITM-ESM	Swapna et al. (2015)[25]
Institute for Numerical Mathematics	INM	INM-CM4	Volodin et al. (2010)[26]
		INM-CM4-8,<br>INM-CM5-0	Volodin et al. (2019)[27]<br>Volodin et al. (2017)[28]
Institut Pierre-Simon Laplace	IPSL	IPSL-CM5A-LR,<br>IPSL-CM5A-MR,<br>IPSL-CM5B-LR	Dufresne et al. (2013)[29]
		IPSL-CM6A-LR	Lurton et al. (2020)[30]
Japan Agency for Marine-Earth Science and Technology, Atmosphere and Ocean Research Institute (The University of Tokyo), and National Institute for Environmental Studies	MIROC	MIROC5,<br>MIROC-ESM,<br>MIROC-ESM-CHEM	Watanabe et al. (2010)[31]<br>Watanabe et al. (2011)[32]
		MIROC6,<br>MIROC-ES2L	Tatebe et al. (2019)[33]<br>Hajima et al. (2020)[34]
Max-Planck-Institut für Meteorologie	MPI-M	MPI-ESM-LR,<br>MPI-ESM-MR	Raddatz et al. (2007)[35]; Marsland et al. (2003)[36]
		MPI-ESM1-2-HR,<br>MPI-ESM1-2-LR	Müller et al. (2018)[37]<br>Mauritsen et al. (2019)[38]
Meteorological Research Institute	MRI	MRI-CGCM3,<br>MRI-ESM1	Yukimoto et al. (2011)[39]
		MRI-ESM2-0	Yukimoto et al. (2019)[40]
Nanjing University of Information Science and Technology	NUIST	NESM3	Cao et al. (2018)[41]

Norwegian Climate Centre	NCC	NorESM1-M	Bentsen et al. (2013)[42]; Iversen et al. (2013)[43]
		NorESM2-LM, NorESM2-MM	Seland et al. (2020)[44]

For CMIP5, daily output has been archived for the CCSM4, CMCC-CESM, CMCC-CMS, HADGEM2-CC, INM-CM4, MPI-ESM-LR, MPI-ESM-MR, MRI-ESM1 GCM runs and 6 hourly data is available for the other 19 GCM runs.

For CMIP6, daily output has been archived for the CANESM5, CESM2, CESM2-WACCM, CNRM-CM6-1, CNRM-CM6-1-HR, CNRM-ESM2-1, EC-EARTH3-VEG, EC-EARTH3, FGOALS-G3, GFDL-CM4, HADGEM3-GC31-LL, HADGEM3-GC31-MM, INM-CM4-8, INM-CM5-0, IPSL-CM6A-LR, MIROC6, MPI-ESM1-2-HR, MRI-ESM2-0, NORESM2-LM, NORESM2-MM, UKESM1-0-LL GCM runs and 6 hourly data is available for the other 5 GCM runs.

Supplementary Table 2 PFP^{LM} trend results for the reanalyses and CMIP6 GCM output without temperature offset.

Same as Table 1 of the main article, except no temperature offset for the formation of PSCs has been applied to GCM output.

GCM	Time Range	W	S	$S_{PFP-LM} \pm \Delta S_{PFP-LM}$ (d decade^{-1})	p-value for S_{PFP-LM}	$\frac{S_{PFP-LM}}{\Delta S_{PFP-LM}}$	p-value for $\frac{S_{PFP-LM}}{\Delta S_{PFP-LM}}$
BCC-CSM2-MR	1951-2100	150	38	1.36 ± 0.15	2×10^{-3}	9.3	4×10^{-4}
CanESM5	1951-2100	150	38	0.98 ± 0.08	3×10^{-3}	12.1	$< 10^{-6}$
CESM2	1951-2100	107	27	1.94 ± 0.13	0.07	14.5	9×10^{-6}
CESM2-WACCM	1951-2100	150	38	0.48 ± 0.14	0.20	3.4	0.22
CNRM-CM6-1	1951-2100	150	38	2.53 ± 0.45	8×10^{-4}	17.9	$< 10^{-6}$
CNRM-CM6-1-HR	1951-2100	150	38	1.55 ± 0.11	0.04	14.6	$< 10^{-6}$
CNRM-ESM2-1	1951-2100	150	38	2.19 ± 0.17	3×10^{-3}	12.8	$< 10^{-6}$
EC-Earth3	1951-2100	150	38	1.45 ± 0.08	0.17	18.6	8×10^{-5}
EC-Earth3-Veg	1951-2100	150	38	0.94 ± 0.03	0.08	26.9	$< 10^{-6}$
FGOALS-g3	1951-2100	150	38	−0.03 ± 0.25	0.49	−0.1	0.49
GFDL-CM4	1951-2100	150	38	0.71 ± 0.04	0.33	18.9	2×10^{-4}
HadGEM3-GC31-LL	1951-2100	150	38	1.50 ± 0.08	0.04	18.0	$< 10^{-6}$
HadGEM3-GC31-MM	1951-2100	150	38	1.30 ± 0.08	0.07	15.7	$< 10^{-6}$
IITM-ESM	1951-2099	149	37	1.31 ± 0.08	0.29	15.7	6×10^{-4}
INM-CM4-8	1951-2100	150	38	2.62 ± 0.18	3×10^{-3}	15.0	4×10^{-5}
INM-CM5-0	1951-2100	150	38	1.39 ± 0.07	0.12	19.0	$< 10^{-6}$
IPSL-CM6A-LR	1951-2100	150	38	0.56 ± 0.04	0.23	15.7	2×10^{-4}
MIROC6	1951-2100	150	38	1.57 ± 0.09	0.18	18.2	$< 10^{-6}$
MIROC-ES2L	1951-2100	150	38	3.13 ± 0.07	2×10^{-4}	46.2	$< 10^{-6}$
MPI-ESM1-2-HR	1951-2100	150	38	1.10 ± 0.07	2×10^{-3}	15.0	2×10^{-5}
MPI-ESM1-2-LR	1951-2100	150	38	1.51 ± 0.12	1×10^{-3}	12.7	2×10^{-4}
MRI-ESM2-0	1951-2100	150	38	0.73 ± 0.08	6×10^{-4}	9.0	2×10^{-4}
NESM3	1951-2100	150	38	1.10 ± 0.07	0.01	16.0	$< 10^{-6}$
NorESM2-LM	1951-2100	150	38	1.35 ± 0.07	0.16	20.6	4×10^{-5}
NorESM2-MM	1951-2100	150	38	1.16 ± 0.13	0.12	8.8	0.08
UKESM1-0-LL	1951-2100	150	38	1.51 ± 0.11	0.02	13.8	$< 10^{-6}$

Supplementary Table 3 Statistics for Monte-Carlo simulation.

Mean slopes $(\overline{S_{\mathrm{PFP-LM}}})$, mean 1σ uncertainties $(\overline{\Delta S_{\mathrm{PFP-LM}}})$, the ratio of these two terms, as well as the frequency of negative slopes ($f_{S_{\mathrm{PFP-LM}}} < 0$), the mean number of selected fit points ($\bar{k}$), and a minimum criteria for number of fit points (k_{MIN}) for VAS, for fits to one million artificial data sets covering 41 years, using the ISA, MIM, and VAS selection procedures.

Selection Technique	$\overline{S_{\mathrm{PFP-LM}}}$ (d decade^{-1})	$\overline{\Delta S_{\mathrm{PFP-LM}}}$ (d decade^{-1})	$\frac{(\overline{S_{\mathrm{PFP-LM}}})}{(\overline{\Delta S_{\mathrm{PFP-LM}}})}$	$f_{S_{\mathrm{PFP-LM}}} < 0$ (%)	$\bar{k}$	k_{MIN}
ISA	4.50	0.42	10.6	0.2	10	–
MIM	3.95	0.92	4.3	0.04	8	–
VAS	2.28	1.58	1.4	6.9	7.36	3
VAS	2.26	1.56	1.4	6.8	7.39	5
VAS	2.08	1.42	1.5	6.6	7.92	7
VAS*	2.30	1.24	1.8	3.2	10	–

VAS*: Value Above Sigma selection forced to always use 10 points

Supplementary Table 4 PFP^{LM} trend results for the reanalyses and CMIP5 GCM output.

Same as Table 1 of the main article, except results are shown for 27 CMIP5 GCMs.

Reanalysis/GCM	Time Range	W	S	T_{OFFSET} (K)	$S_{PFP-LM} \pm \Delta S_{PFP-LM}$ (d decade^{-1})	p-value for S_{PFP-LM}	$\frac{S_{PFP-LM}}{\Delta S_{PFP-LM}}$	p-value for $\frac{S_{PFP-LM}}{\Delta S_{PFP-LM}}$
BCC-CSM1-1	1951-2100	150	38	–3	0.74 ± 0.07	0.02	11.1	$< 10^{-6}$
BCC-CSM1-1(m)	1951-2100	150	38	–2	0.84 ± 0.08	0.03	10.9	4×10^{-4}
BNU-ESM	1951-2100	150	38	–3	2.42 ± 0.19	$< 10^{-6}$	12.7	$< 10^{-6}$
CanESM2	1951-2100	150	38	3	1.77 ± 0.21	0.04	8.4	0.02
CCSM4	1951-2100	150	38	–4	0.81 ± 0.08	2×10^{-3}	10.1	4×10^{-5}
CMCC-CESM	1951-2100	150	38	–8	-0.96 ± 0.14	0.11	-7.1	0.04
CMCC-CM	1951-2100	149	37	–2	1.14 ± 0.11	0.05	10.1	3×10^{-3}
CMCC-CMS	1951-2100	150	38	4	0.10 ± 0.14	0.39	0.7	0.40
CNRM-CM5	1951-2100	150	38	1	1.74 ± 0.11	0.02	16.0	$< 10^{-6}$
GFDL-CM3	1951-2100	150	38	0	1.61 ± 0.07	2×10^{-4}	23.0	$< 10^{-6}$
GFDL-ESM2G	1951-2100	150	38	4	1.48 ± 0.12	5×10^{-3}	12.0	$< 10^{-6}$
GFDL-ESM2M	1951-2100	150	38	4	1.53 ± 0.09	6×10^{-3}	16.6	$< 10^{-6}$
GISS-E2-H	1951-2100	149	37	5	1.60 ± 0.07	0.01	23.9	$< 10^{-6}$
GISS-E2-R	1951-2100	149	37	5	1.85 ± 0.11	9×10^{-4}	16.9	$< 10^{-6}$
HadGEM2-CC	1951-2100	150	38	3	1.47 ± 0.12	0.02	12.5	4×10^{-5}
INM-CM4	1951-2100	150	38	5	4.46 ± 0.18	0.02	25.4	$< 10^{-6}$
IPSL-CM5A-LR	1951-2100	150	38	1	1.05 ± 0.09	0.12	11.3	2×10^{-3}
IPSL-CM5A-MR	1951-2100	150	38	2	1.71 ± 0.10	0.02	17.2	$< 10^{-6}$
IPSL-CM5B-LR	1951-2100	150	38	0	1.41 ± 0.14	0.03	9.9	4×10^{-3}
MIROC5	1951-2100	150	38	2	3.57 ± 0.07	7×10^{-4}	50.2	$< 10^{-6}$
MIROC-ESM	1951-2100	150	38	2	2.37 ± 0.11	7×10^{-4}	20.8	$< 10^{-6}$
MIROC-ESM-CHEM	1951-2100	150	38	2	2.71 ± 0.13	8×10^{-5}	21.1	$< 10^{-6}$
MPI-ESM-LR	1951-2100	150	38	0	0.05 ± 0.19	0.48	0.3	0.49
MPI-ESM-MR	1951-2100	150	38	0	0.52 ± 0.11	0.04	4.8	0.03
MRI-CGCM3	1951-2100	150	38	0	0.99 ± 0.08	0.03	11.9	2×10^{-4}
MRI-ESM1	1951-2100	150	38	1	0.95 ± 0.09	4×10^{-3}	10.3	9×10^{-4}
NorESM1_M	1951-2100	150	38	–1	1.63 ± 0.12	5×10^{-3}	14.0	$< 10^{-6}$

Supplementary References

1. Hersbach, H. *et al.* The ERA5 global reanalysis. *Q. J. R. Meteorol. Soc.* **146**, 1999–2049 (2020).
2. Kivi, R., Kyrö, E., Turunen, T., Ulich, T. & Turunen, E. Atmospheric trends above Finland: II. Troposphere and stratosphere. *Geophysica* **35**, 71–85 (1999).
3. Wu, T. *et al.* An overview of BCC climate system model development and application for climate change studies. *J. Meteorol. Res.* **28**, 34–56 (2014).
4. Wu, T. *et al.* The Beijing Climate Center Climate System Model (BCC-CSM): the main progress from CMIP5 to CMIP6. *Geosci. Model Dev.* **12**, 1573–1600 (2019).
5. Ji, D. *et al.* Description and basic evaluation of Beijing Normal University Earth System Model (BNU-ESM) version 1. *Geosci. Model Dev.* **7**, 2039–2064 (2014).
6. Swart, N. C. *et al.* The Canadian Earth System Model version 5 (CanESM5.0.3). *Geosci. Model Dev.* **12**, 4823–4873 (2019).
7. Gent, P. R. *et al.* The Community Climate System Model Version 4. *J. Clim.* **24**, 4973–4991 (2011).
8. Danabasoglu, G. *et al.* The Community Earth System Model Version 2 (CESM2). *J. Adv. Model. Earth Syst.* **12**, e2019MS001916 (2020).
9. Fogli, P. G. *et al. INGV-CMCC Carbon (ICC): A Carbon Cycle Earth System Model. SSRN Electronic Journal*, doi:10.2139/ssrn.1517282 (2011).
10. Vichi, M. *et al.* Global and regional ocean carbon uptake and climate change: sensitivity to a substantial mitigation scenario. *Clim. Dyn.* **37**, 1929–1947 (2011).
11. Scoccimarro, E. *et al.* Effects of tropical cyclones on ocean heat transport in a high-resolution coupled general circulation model. *J. Clim.* **24**, 4368–4384 (2011).
12. Voldoire, A. *et al.* The CNRM-CM5.1 global climate model: description and basic evaluation. *Clim. Dyn.* **40**, 2091–2121 (2013).
13. Voldoire, A. *et al.* Evaluation of CMIP6 DECK experiments with CNRM-CM6-1. *J. Adv. Model. Earth Syst.* **11**, 2177–2213 (2019).
14. (EC-Earth), E.-E. C. EC-Earth-Consortium EC-Earth3 model output prepared for CMIP6 CMIP. doi:10.22033/ESGF/CMIP6.181 (2019).
15. (EC-Earth), E.-E. C. EC-Earth-Consortium EC-Earth3-Veg model output prepared for CMIP6 ScenarioMIP. doi:10.22033/ESGF/CMIP6.727 (2019).
16. Li, L. CAS FGOALS-g3 model output prepared for CMIP6 CMIP. doi:10.22033/ESGF/CMIP6.1783 (2019).
17. Donner, L. J. *et al.* The dynamical core, physical parameterizations, and basic simulation characteristics of the atmospheric component AM3 of the GFDL global coupled model CM3. *J. Clim.* **24**, 3484–3519 (2011).
18. Dunne, J. P. *et al.* GFDL's ESM2 global coupled climate-carbon earth system models. Part I: Physical formulation and baseline simulation characteristics. *J. Clim.* **25**, 6646–6665 (2012).
19. Dunne, J. P. *et al.* GFDL's ESM2 global coupled climate-carbon earth system models. Part II: Carbon system formulation and baseline simulation characteristics. *J. Clim.* **26**, 2247–2267 (2013).
20. Held, I. M. *et al.* Structure and performance of GFDL's CM4.0 climate model. *J. Adv. Model. Earth Syst.* **11**, 3691–3727 (2019).
21. Schmidt, G. A. *et al.* Configuration and assessment of the GISS ModelE2 contributions to the CMIP5 archive. *J. Adv. Model. Earth Syst.* **6**, 141–184 (2014).
22. Martin, G. M. *et al.* The HadGEM2 family of Met Office Unified Model climate configurations. *Geosci. Model Dev.* **4**, 723–757 (2011).
23. Collins, W. J. *et al.* Development and evaluation of an Earth-System model – HadGEM2. *Geosci. Model Dev.* **4**, 1051–1075 (2011).
24. Andrews, T. *et al.* Forcings, feedbacks, and climate sensitivity in HadGEM3-GC3.1 and UKESM1. *J. Adv. Model. Earth Syst.* **11**, 4377–4394 (2019).
25. Swapna, P. *et al.* The IITM Earth system model: transformation of a seasonal prediction model

to a long-term climate model. *Bull. Am. Meteorol. Soc.* **96**, 1351–1367.
26. Volodin, E. M., Dianskii, N. A. & Gusev, A. V. Simulating present-day climate with the INMCM4.0 coupled model of the atmospheric and oceanic general circulations. *Izv. Atmos. Ocean. Phys.* **46**, 414–431 (2010).
27. Volodin, E. *et al.* INM INM-CM4-8 model output prepared for CMIP6 CMIP. doi:10.22033/ESGF/CMIP6.1422 (2019).
28. Volodin, E. M. *et al.* Simulation of the present-day climate with the climate model INMCM5. *Clim. Dyn.* **49**, 3715–3734 (2017).
29. Dufresne, J.-L. *et al.* Climate change projections using the IPSL-CM5 Earth System Model: from CMIP3 to CMIP5. *Clim. Dyn.* **40**, 2123–2165 (2013).
30. Lurton, T. *et al.* Implementation of the CMIP6 forcing data in the IPSL-CM6A-LR model. *J. Adv. Model. Earth Syst.* **12**, e2019MS001940 (2020).
31. Watanabe, M. *et al.* Improved climate simulation by MIROC5: Mean states, variability, and climate sensitivity. *J. Clim.* **23**, 6312–6335 (2010).
32. Watanabe, S. *et al.* MIROC-ESM 2010: model description and basic results of CMIP5-20c3m experiments. *Geosci. Model Dev.* **4**, 845–872 (2011).
33. Tatebe, H. *et al.* Description and basic evaluation of simulated mean state, internal variability, and climate sensitivity in MIROC6. *Geosci. Model Dev.* **12**, 2727–2765 (2019).
34. Hajima, T. *et al.* Development of the MIROC-ES2L Earth system model and the evaluation of biogeochemical processes and feedbacks. *Geosci. Model Dev.* **13**, 2197–2244 (2020).
35. Raddatz, T. J. *et al.* Will the tropical land biosphere dominate the climate–carbon cycle feedback during the twenty-first century? *Clim. Dyn.* **29**, 565–574 (2007).
36. Marsland, S. J., Haak, H., Jungclaus, J. H., Latif, M. & Röske, F. The Max-Planck-Institute global ocean/sea ice model with orthogonal curvilinear coordinates. *Ocean Model.* **5**, 91–127 (2003).
37. Müller, W. A. *et al.* A higher-resolution version of the Max Planck Institute Earth System Model (MPI-ESM1.2-HR). *J. Adv. Model. Earth Syst.* **10**, 1383–1413 (2018).
38. Mauritsen, T. *et al.* Developments in the MPI-M Earth System Model version 1.2 (MPI-ESM1.2) and its response to increasing CO_2. *J. Adv. Model. Earth Syst.* **11**, 998–1038 (2019).
39. Yukimoto, S. *et al. Meteorological Research Institute-Earth System Model Version 1 (MRI-ESM1) - Model Description*. doi:10.11483/mritechrepo.64 (2011).
40. Yukimoto, S. *et al.* The meteorological research institute Earth system model version 2.0, MRI-ESM2.0: Description and basic evaluation of the physical component. *J. Meteorol. Soc. Japan* **97**, 931–965 (2019).
41. Cao, J. *et al.* The NUIST Earth System Model (NESM) version 3: description and preliminary evaluation. *Geosci. Model Dev.* **11**, 2975–2993 (2018).
42. Bentsen, M. *et al.* The Norwegian Earth System Model, NorESM1-M – Part 1: Description and basic evaluation of the physical climate. *Geosci. Model Dev.* **6**, 687–720 (2013).
43. Iversen, T. *et al.* The Norwegian Earth System Model, NorESM1-M – Part 2: Climate response and scenario projections. *Geosci. Model Dev.* **6**, 389–415 (2013).
44. Seland, Ø. *et al.* Overview of the Norwegian Earth System Model (NorESM2) and key climate response of CMIP6 DECK, historical, and scenario simulations. *Geosci. Model Dev.* **13**, 6165–6200 (2020).
